# An optimized genome-wide, virus-free CRISPR screen for mammalian cells

**DOI:** 10.1016/j.crmeth.2021.100062

**Published:** 2021-08-04

**Authors:** Kai Xiong, Karen Julie la Cour Karottki, Hooman Hefzi, Songyuan Li, Lise Marie Grav, Shangzhong Li, Philipp Spahn, Jae Seong Lee, Ildze Ventina, Gyun Min Lee, Nathan E. Lewis, Helene Faustrup Kildegaard, Lasse Ebdrup Pedersen

**Affiliations:** 1 The Novo Nordisk Foundation Center for Biosustainability, Technical University of Denmark, Lyngby, Denmark; 2 The Novo Nordisk Foundation Center for Biosustainability, University of California at San Diego, La Jolla, CA, USA; 3 Department of Molecular Science and Technology, Ajou University, Suwon 16499, Republic of Korea; 4 Department of Biological Sciences, KAIST, Daejeon, Republic of Korea; 5 Department of Pediatrics, University of California at San Diego, La Jolla, CA, USA; 6 Department of Bioengineering, University of California at San Diego, La Jolla, CA, USA; 7 Department of Bioengineering, Technical University of Denmark, Lyngby, Denmark; 8 Lead contact

## Abstract

Pooled CRISPR screens have been widely applied to mammalian and other organisms to elucidate the interplay between genes and phenotypes of interest. The most popular method for delivering the CRISPR components into mammalian cells is lentivirus based. However, because lentivirus is not always an option, virus-free protocols are starting to emerge. Here, we demonstrate an improved virus-free, genome-wide CRISPR screening platform for Chinese hamster ovary cells with 75,488 gRNAs targeting 15,028 genes. Each gRNA expression cassette in the library is precisely integrated into a genomic landing pad, resulting in a very high percentage of single gRNA insertions and minimal clonal variation. Using this platform, we perform a negative selection screen on cell proliferation that identifies 1,980 genes that affect proliferation and a positive selection screen on the toxic endoplasmic reticulum stress inducer, tunicamycin, that identifies 77 gene knockouts that improve survivability.

## In brief

CRISPR knockout screening has mostly been performed by using viruses to deliver the required components into cells. In this paper, Xiong et al. demonstrate a virus-free approach that reduces noise and broadens access to CRISPR-based screens.

## Graphical abstract

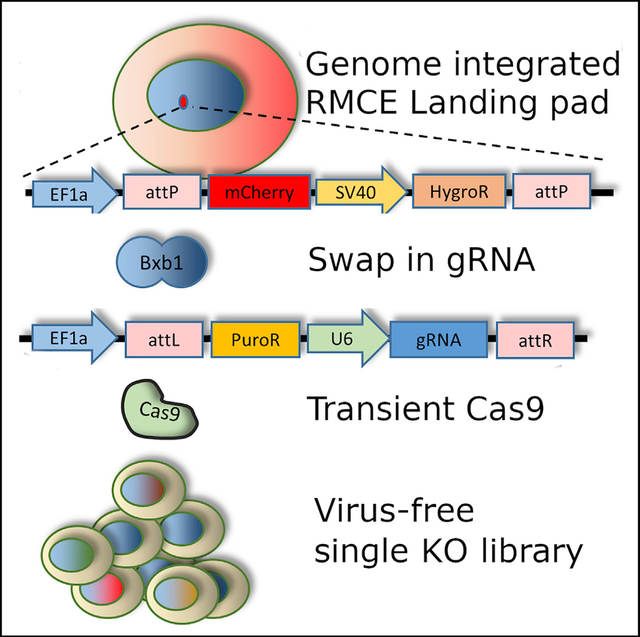

## INTRODUCTION

CRISPR screens have been widely applied to decipher mammalian gene function at the genome scale, but most rely on lentiviral delivery methods (Adamson et al., 2016; Joung et al., 2017; Liu et al., 2018). However, lentivirus is not always an option. Working with lentivirus requires specialized facilities as well as trained personnel, which are not available in all laboratories. In some industrial and medical facilities, it is likewise considered an increased risk to work with live viruses. As we demonstrate in this paper, a recombinase-mediated cassette exchange (RMCE)-based, virus-free (VF) method can not only eliminate those concerns, but also result in less noisy data. RMCE is a method in which one can move a piece of DNA, for example, from a plasmid, into a pre-established genome landing pad without causing large-scale disruptions that can occur with CRISPR-mediated targeted insertion.

VF CRISPR screens are starting to emerge using various alternative strategies to perform the screen and detect hits. Examples are replacing an integrated dummy guide RNA (gRNA) with a pooled library of gRNAs by using homologous recombination (Rajagopal et al., 2016) or performing whole-genome sequencing to detect mutations caused by transient CRISPR expression (Kim et al., 2017). However, these strategies suffer from drawbacks. The method based on homologous recombination reports that the efficiency of the gRNA cassette integration is much lower than lentiviral integration efficiency, and it cannot be enriched by selection because of a risk of random integration of promoter-less gRNA that complicates “hit detection.” This makes it difficult to perform very large screens because of the very large number of cells needed (Rajagopal et al., 2016). The second method, applying whole-genome sequencing to detect CRISPR-caused mutations in a pool of cells, is in our opinion more promising, but reliably linking such mutations to phenotype is not trivial, especially when using genomically unstable cell lines such as HEK, HeLa, other cancer cell lines, or Chinese hamster ovary (CHO) cells and, in addition, full genome sequencing will need to be performed at a very high depth to reliably detect any effect less than “extreme” (Kim et al., 2017).

In this study, we present a new VF CRISPR screening platform and demonstrate it in CHO cells, the model of choice for producing pharmaceutical proteins (Walsh, 2018). We apply this platform to investigate key phenotypes in protein production contexts, namely, cell proliferation and ER stress resistance.

We obtain an in-cell library that covers 99.95% of our 75,488 gRNA large library with 92.5% of the cells containing exactly one gRNA. We show that this VF approach has low noise and works for both depletion and enrichment studies.

## RESULTS

### Design of the virus-free, genome-wide CRISPR pooled screening platform

**Figure 1.**
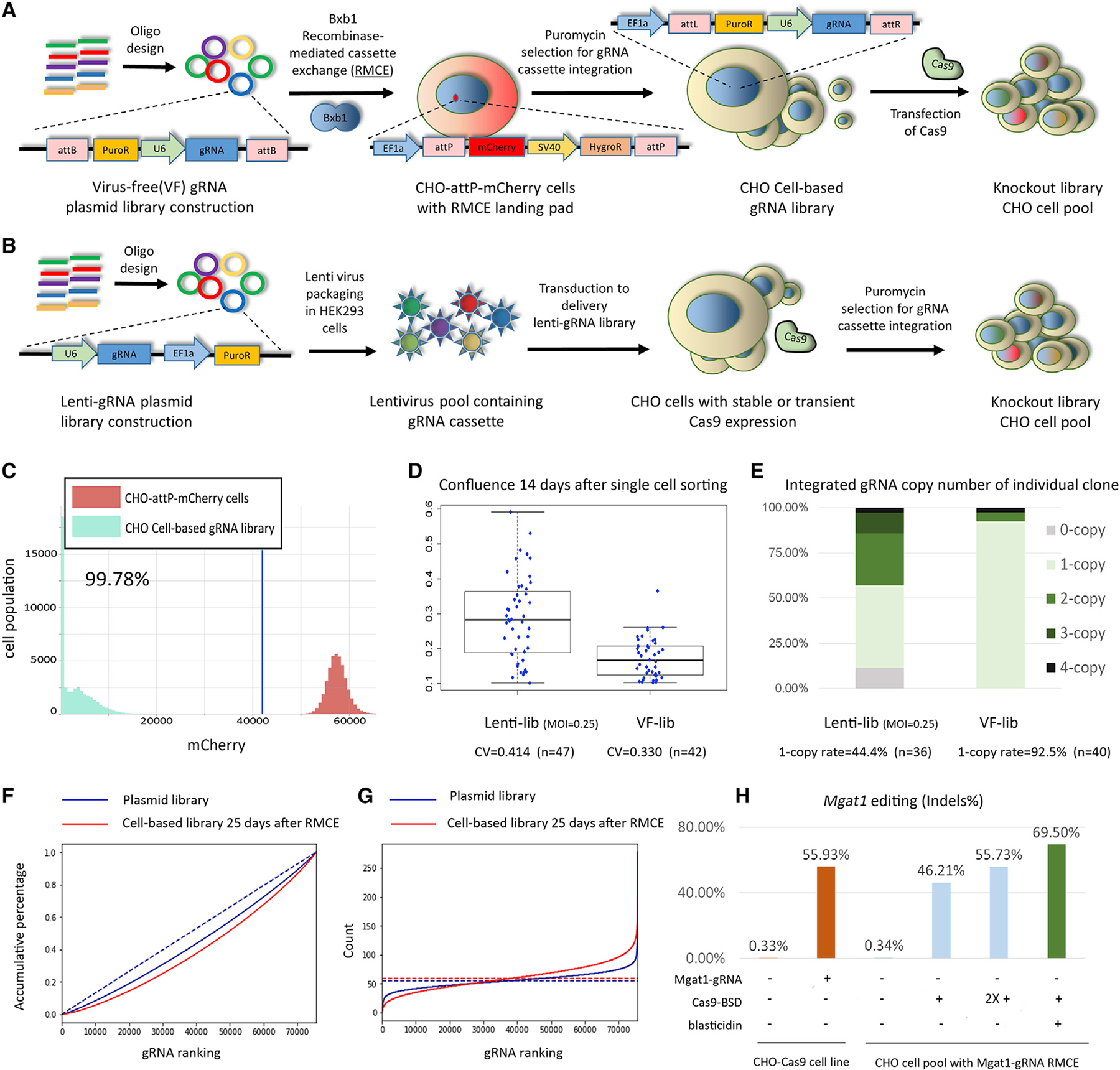
Establishing a virus-free (VF) CRISPR screen platform (A and B) Shown are a (A) VF CRISPR screen and a (B) lentiviral CRISPR screen. A genome-wide VF library was designed, containing 74,617 gRNAs targeting 18,353 expressed genes in CHO-S cells with 1,000 non-targeting control gRNAs. The gRNA expression cassette was precisely integrated into the working cell line by Bxb1 RMCE. A promoter-less puromycin-resistance gene (PuroR) in the RMCE donor DNA enables the precise integration of a single gRNA expression cassette. The lentiviral library contained 15,645 gRNAs targeting 2,599 genes with 1,000 non-targeting control gRNAs (lentiviral library). (C) In the VF library, the cell pool containing the gRNA library was enriched after 25 days of puromycin selection. The mCherry cassette was finally replaced by the gRNA expression cassette. (D) Before Cas9 was delivered, the cell clone variance in the lentiviral library and VF library was compared by measuring the colony confluence 14 days after single-cell sorting into 96-well plates. CV indicates coefficient of variation. (E and F) The (E) gRNA copy number in the lentiviral library and VF library was further detected by qPCR. gRNA coverage of the VF library was calculated in (F). Shown are the cumulative percentage of reads in the plasmid library after vector construction (blue solid line) and in the cell-based library 25 days after RMCE (red solid line). Dashed line, ideal model in gRNA library design. (G) The number of reads per gRNA in the plasmid library after vector construction (blue solid line) and in the cell-based library 25 days after RMCE (red solid line). Dashed blue line, ideal model in plasmid library (mean of reads). Dashed red line, ideal model in cell-based library 25 days after RMCE (mean of reads). (H) Testing of Cas9 delivery method by using *Mgat1* as the target gene. After a cell pool was established with RMCE by using gRNA targeting *Mgat1* (Mgat1-gRNA), the transient Cas9-BSD vector was transfected. The Cas9-transfected cells were enriched by treatment with blasticidin. The *Mgat1* editing efficiency was verified by NGS. Transfection of one or two rounds (2X +) of Cas9 in the CHO cell pool with RMCE of Mgat1 was set as control.

Using 1,558 RNA-sequencing (RNA-seq) samples from different CHO cell lines and culture conditions, we selected ~15,000 expressed genes from the CHO genome (Rupp et al., 2018). Then, we designed gRNAs against the coding sequences of these genes (Table S1). These gRNAs were used to build a genome-wide VF library that contained 75,488 unique gRNAs, of which 72,149 target 15,028 genes, 2,218 target intergenic regions, 1,051 are non-targeting (NT) gRNAs, and finally 70 gRNAs target multiple genes (Figure 1A, Table S1).

**Figure 2.**
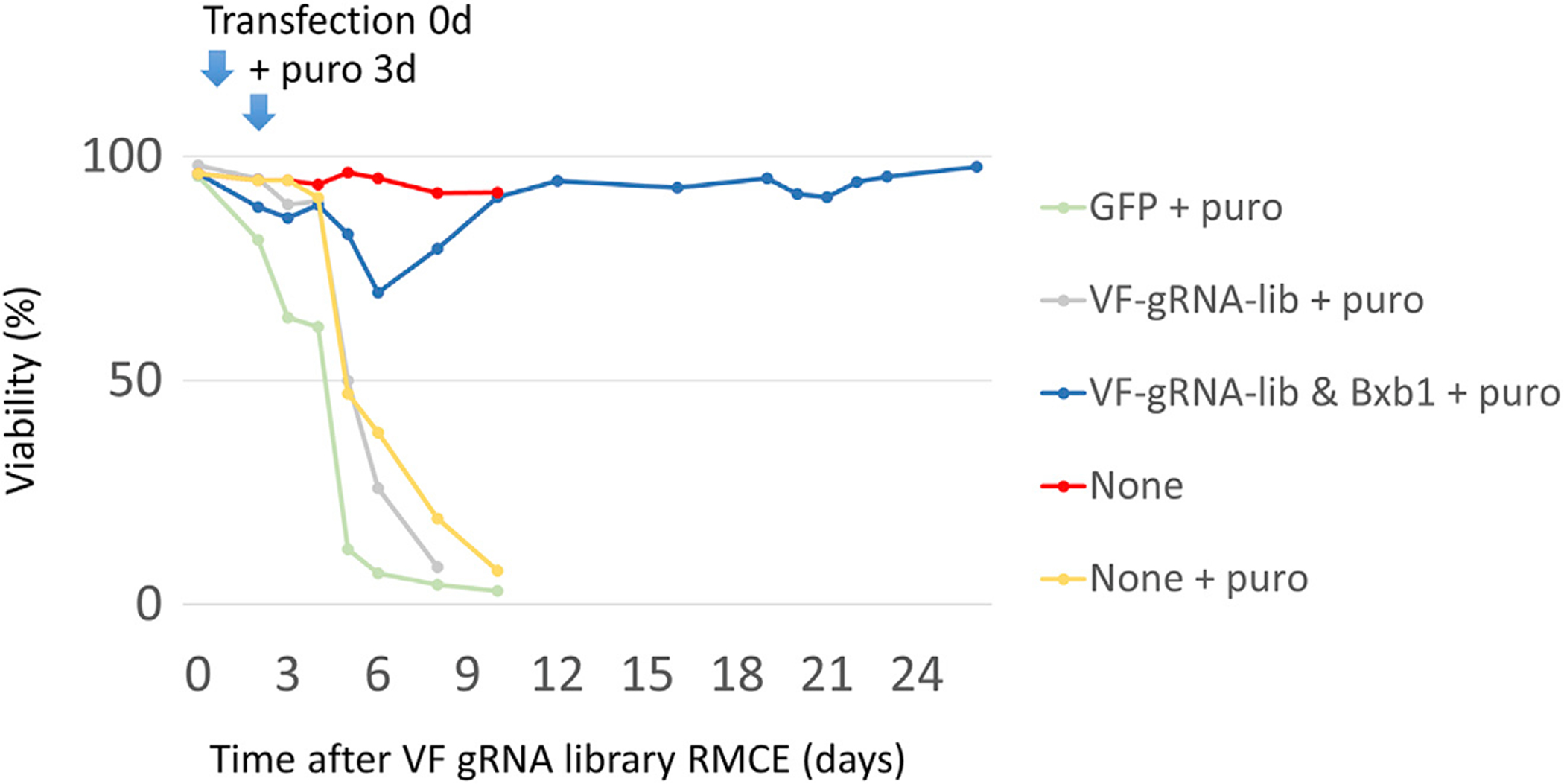
The cell viability during the process of establishing a VF cell-based gRNA library CHO-attp-mCherry cells with RMCE landing pads were co-transfected with Bxb1 recombinase and the VF gRNA library. Three days after transfection, cells were selected with 10 μg/mL puromycin for around 20 days. Cells transfected with GFP (GFP + puro) or without transfection (None + puro) were set as controls for puromycin selection. Cells transfected with only the VF gRNA library and without Bxb1 recombinase (VF-gRNA-lib + puro) confirm the limited random integration of gRNA cassettes. Non-treated cells (None) were used as a control.

In the VF screening platform, we used Bxb1 RMCE to precisely integrate a single gRNA expression cassette. We made a CHO-S cell line with a Bxb1 RMCE landing pad that contains an EF1a promoter followed by a recombinase target site (attP), an mCherry expression unit, and an SV40 promoter driving expression of a hygromycin resistance gene (HygroR) followed by the second recombinase target site (mutant attP). The integration site of this landing pad was previously selected as an active site surrounded by highly expressed genes (Petersen et al., 2018; Pristovšek et al., 2019; Xiong et al., 2019). This cell line is termed CHO-attp-mCherry. In the donor gRNA library plasmid, the gRNA expression cassette for RMCE is flanked by recombinase target sites (attB and mutant attB). To allow for enrichment of the integrated gRNA while also preventing enrichment of randomly integrated gRNAs, we placed a promoter-less puromycin resistance gene (PuroR) within the recombination site so that the PuroR gene is expressed only after correct insertion into the landing pad with the EF1a promoter upstream. Lack of mCherry expression thus indicates a successful RMCE of the gRNA cassette. After transfection with the Bxb1 recombinase plasmid and gRNA library plasmids, ~4% of the cells demonstrated successful RMCE (Figure S1). The RMCE-positive cells were then further enriched by puromycin (10 μg/mL) selection for 14–20 days (Figure 2). Twenty-five days after gRNA RMCE, the RMCE cell population was fully enriched, as evidenced by 99.78% of the cells being negative for mCherry (Figure 1C).

### Spurious gene editing is reduced by using transiently expressed Cas9

**Figure 3.**
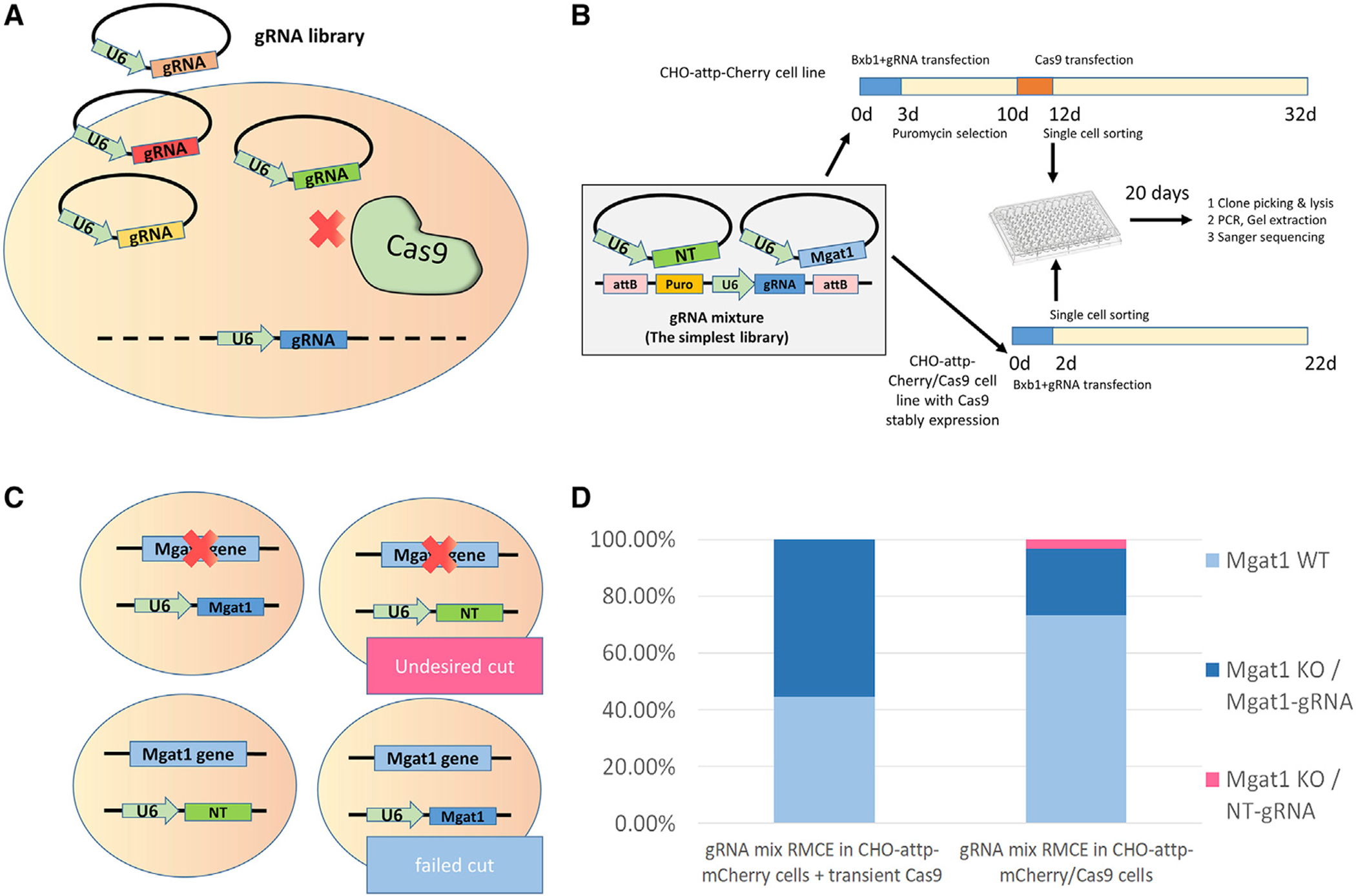
Detection of spurious gene editing in CRISPR (A) Illustration of undesired spurious gene editing caused by the transfected gRNA library plasmid but not by the integrated gRNA cassette. (B) Experimental design to compare the effects of spurious gene editing by transfected gRNA plasmid. The mixture of gRNAs containing Mgat1-gRNA and NT-gRNA were co-transfected with Bxb1 into CHO-attP-mCherry cells, and 10 days afterward the Cas9 was transfected. The control was set as Bxb1 RMCE of the gRNA mixture in CHO-attP-mCherry/Cas9 cells in which Cas9 is stably expressed. Single cells from these two cell pools were sorted to form colonies for the following genomic DNA extraction. (C) The gene editing and gRNA integration patterns were compared between the two protocols in (B). (D) Percentage of different gene editing and gRNA integration patterns in two protocols: gRNA mix RMCE in CHO-attP-mCherry cells + transient Cas9 (n = 8) and gRNA mix RMCE in CHO-attP-mCherry/Cas9 cells (n = 30).

Spurious gene editing can occur by transient expression of gRNAs from plasmids after transfection of the gRNA library if Cas9 is simultaneously expressed (Figure 3A). To overcome this issue, instead of using a stable Cas9-expressing cell line, we used transient Cas9 expression in the gRNA RMCE cell pool, introduced after cell-division-based dilution of non-integrated gRNA plasmids. To confirm that spurious gene editing was avoided using this strategy, we performed a test to compare the transient strategy with a cell line stably expressing Cas9. gRNA targeting *Mgat1* (Mgat1-gRNA) and an NT-gRNA were mixed at the ratio 1:1. This mixture of gRNAs was co-transfected with Bxb1 into CHO-attp-mCherry cells. Ten days after transfection of the gRNA, the CHO-attp-mCherry cells were transiently transfected with Cas9, and meanwhile, the mixture of gRNAs was co-transfected with Bxb1 into CHO cells stably expressing Cas9 (CHO-attp-mCherry/Cas9). Two days later, both cell line populations were single-cell sorted into 96-well plates (Figure 3B). We then tested the cells for *Mgat1* gene editing and gRNA integration in the landing pad by Sanger sequencing. Spurious gene editing would be a case in which *Mgat1* was edited but the NT-gRNA was integrated in the landing pad (Figure 3C). The sequencing analysis confirmed that there is no spurious gene editing when using transient Cas9, whereas undesired spurious editing was found in the stable Cas9 cells, presumably because of gRNA transcribed from unintegrated gRNA plasmids (Figure 3D). We thus successfully avoided such undesired and hard-to-detect spurious gene editing. Knowing this, in the following library establishment, we transfected Cas9 25 days after transfecting the gRNA RMCE system to allow sufficient time for dilution/degradation of unintegrated gRNA expression plasmids.

### Precise integration of gRNA expression cassette results in lower clonal variance in cell population and achieves high library coverage

While constructing and testing the VF library, we simultaneously ran experiments by using a previously established lentiviral library (Figure 1B) to verify that our VF library gave similar results.

We hypothesized that the targeted integration of gRNA might lead to less clonal variation than the randomly integrated gRNA in the lentiviral library. To investigate the effect of gRNA integration methods on the phenotype of CHO cells, before Cas9 delivery, we analyzed the variance of cell proliferation in the lentiviral library and VF library by measuring the colony confluence 14 days after single-cell sorting into 96-well plates (Figure 1D). The cell population resulting from lentiviral library transduction showed higher variation across cells than the VF library population. We hypothesize that this higher clone variance in the lentiviral library population is caused by random, and potentially multiple, genomic integrations of gRNA.

To verify the desired single-copy integration of gRNA cassettes, we used qPCR to determine the copy number of genome-integrated gRNA cassettes in the VF RMCE cells and compared with the lentiviral library. Using VF RMCE, 92.5% of the cells showed single integration of gRNA cassettes, whereas the number of integration events in the lentiviral library followed a Poisson distribution (Figure 1E). Our previous study of the lentiviral library also demonstrated that it is common to have multiple different types of gRNAs in the cells that have multiple gRNA integrations. In summary, the VF integration results in low clone-to-clone variation in the cell pool before performing the screen.

The coverage of the gRNA library after integration was as high (99.95%) in the cells as in the plasmid pool (99.98%), and showed an even distribution of gRNAs (skew ratio [Konermann et al., 2015; Joung et al., 2017] = 1.85 and 2.84 in the plasmid- and cell-based libraries, respectively, Figures 1F and 1G).

### Enriching for Cas9-transfected cells results in a high gene editing rate

To identify an efficient method to deliver Cas9, we tested a vector expressing Cas9 together with a blasticidin resistance gene (BSD) in CHO cells followed by treatment with blasticidin to kill the cells without transient Cas9 expression. Four days of blasticidin treatment was sufficient to kill untransfected cells (Figure S2). We verified this method via next-generation sequencing (NGS) by using the *Mgat1* gene as a model. In the blasticidin-selected Cas9-transfected CHO cells with RMCE of gRNA targeting *Mgat1* (Mgat1-gRNA), *Mgat1* gene editing efficiency was ~70%, which is higher than the efficiency via transfection of 1 or 2 rounds of Cas9-BSD without blasticidin selection. Also, the efficiency was higher than transient transfection of the gRNA into a CHO cell line with constitutive Cas9 expression (CHO-Cas9, Figure 1H). With the protocol for enriching Cas9-transfected cells now validated, we transfected the Cas9-BSD into the cells with the integrated gRNA library, and we subsequently followed with blasticidin selection to establish the final knockout (KO) CHO cell pool (Figures S2C and S2D).

### Genes essential for cell proliferation are identified in the VF CRISPR screen

**Figure 4.**
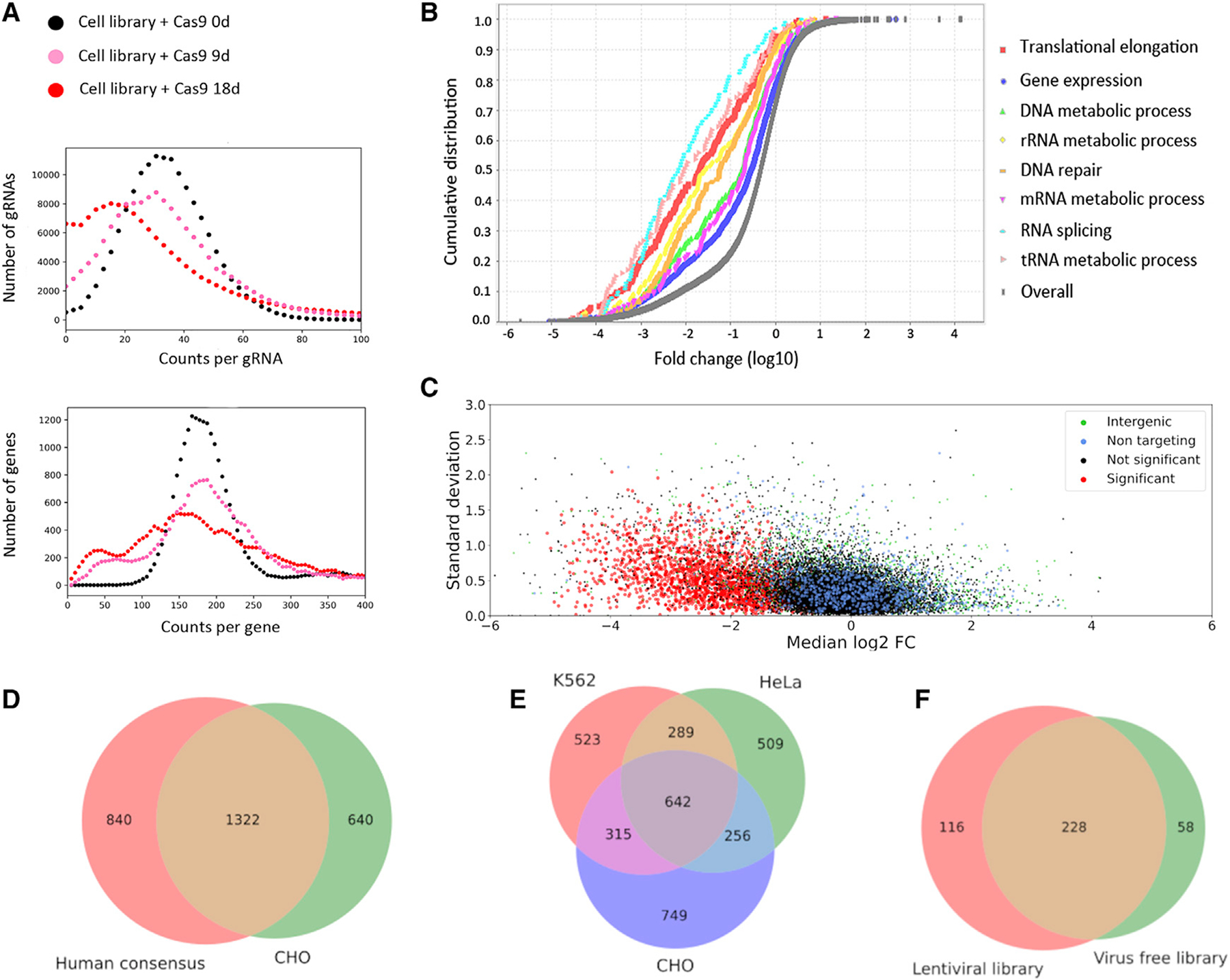
Genome-wide VF CRISPR screen to identify essential genes in CHO cells (A) gRNA read distribution of targeted genes after Cas9 transfection in the cell-based gRNA library. (B) Selected biological processes significantly depleted in CHO cells 18 days after Cas9 transfection in cell-based gRNA library (p < 0.05). (C–E) Shown are the (C) fold change (median of log_2_) and the standard deviation of gRNAs among three biological screening replicates. The significantly depleted gRNAs were classified as gRNAs targeting essential genes (highlighted in red). Venn diagrams of candidate essential genes in CHO cells and the reported essential genes (D) in human and (E) in K562 and HeLa cells. (F) Overlap of essential genes between VF growth screen and lentiviral growth screen.

Nine and eighteen days after Cas9 transfection, the genomic DNA from the cell pool was harvested for NGS analysis to measure the gRNA depletion. The percentage of depleted gRNAs increased after Cas9 transfection into the cell-based gRNA library (Figure 4A). Eighteen days after Cas9 transfection in the cell-based gRNA library, gene set enrichment analysis revealed significantly depleted genes in fundamental cellular processes: genes involved in DNA replication and repair, gene expression, RNA processing and splicing, and protein translation (Mi et al., 2019a, 2019b) (Figure 4B, Table S3).The significant depletion of gRNAs targeting 1,980 genes was shown in three biologically independent samples compared with NT-gRNAs (Figure 4C and Table S4), indicating that these genes are likely essential to cell viability and/or proliferation under our culture conditions.

Using the essential gene database (www.essentialgene.org) (Luo et al., 2014), we compared our results to previous findings (Figures 4D and 4E, Table S5). Using all 20 human datasets from the essential gene database, we created a consensus set of essential genes consisting of all genes present in at least five of those studies. Compared with this consensus set, we found that 1,322 genes (~47%) are overlapping (Figure 4D). In addition, we analyzed previous pooled CRISPR KO screens in two human cell lines and found that K562 cells (Wang et al., 2017b) and HeLa cells (Hart et al., 2015) have similar amounts of overlap with each other as they do with our CHO data (~37% between K562 and HeLa, ~35% between K562 and CHO, ~33% between HeLa and CHO) (Figure 4E, Table S5). From this we conclude that our VF CRISPR screen method can identify essential genes via gRNA depletion analysis as well as other libraries.

We also wanted to see how our results compared with a similar screen performed on our smaller lentivirus-based library. The overall fold change of targeted genes in both screens showed high correlation after Cas9 induction (Figure S3). The VF library and the lentiviral library have an overlap of 1,553 targeted genes, and of these, 344 were found to be significantly enriched in the lentiviral library and 286 were found in the VF library (Figure 4F). Of those genes, 228 (57%) were identical, much higher than the overlap between the two human cell lines K562 and HeLa (~37%).

In summary, we identified 1,980 genes that have a negative impact on cell proliferation and/or viability. The list includes genes coding for key catalytic enzymes such as fumarate hydra-tase, NADH dehydrogenase, and fatty acid synthase (Tables S3 and S4). This information can be used to further understand cell metabolism and proliferation.

### Genes sensitive to induced ER stress are identified in the VF CRISPR screen

Although many proteins, most notably antibodies, are readily produced in CHO cells, some proteins are currently difficult or impossible to produce. Such proteins are termed “difficult-to-express” (DTE) or “difficult-to-produce” (DTP). Some of these proteins result in high levels of unfolded proteins, which leads to the activation of the unfolded protein response (UPR) and ultimately leads to apoptosis (Prashad and Mehra, 2015). Some investigations indicate that reducing the UPR-mediated ER stress in CHO cells can improve protein productivity (Tigges and Fussenegger, 2006; Borth et al., 2008; Mohan and Lee, 2010; Le Fourn et al., 2014). As a simple demonstration of an enrichment screen, we used the ER-stress-inducing compound tunicamycin (TM) to simulate a DTE/DTP production situation. CHO cells grown in TM stop proliferating and ultimately die. By applying the VF CRISPR screen platform, we identified genes that mitigate the effect of TM on CHO cells.

**Figure 5.**
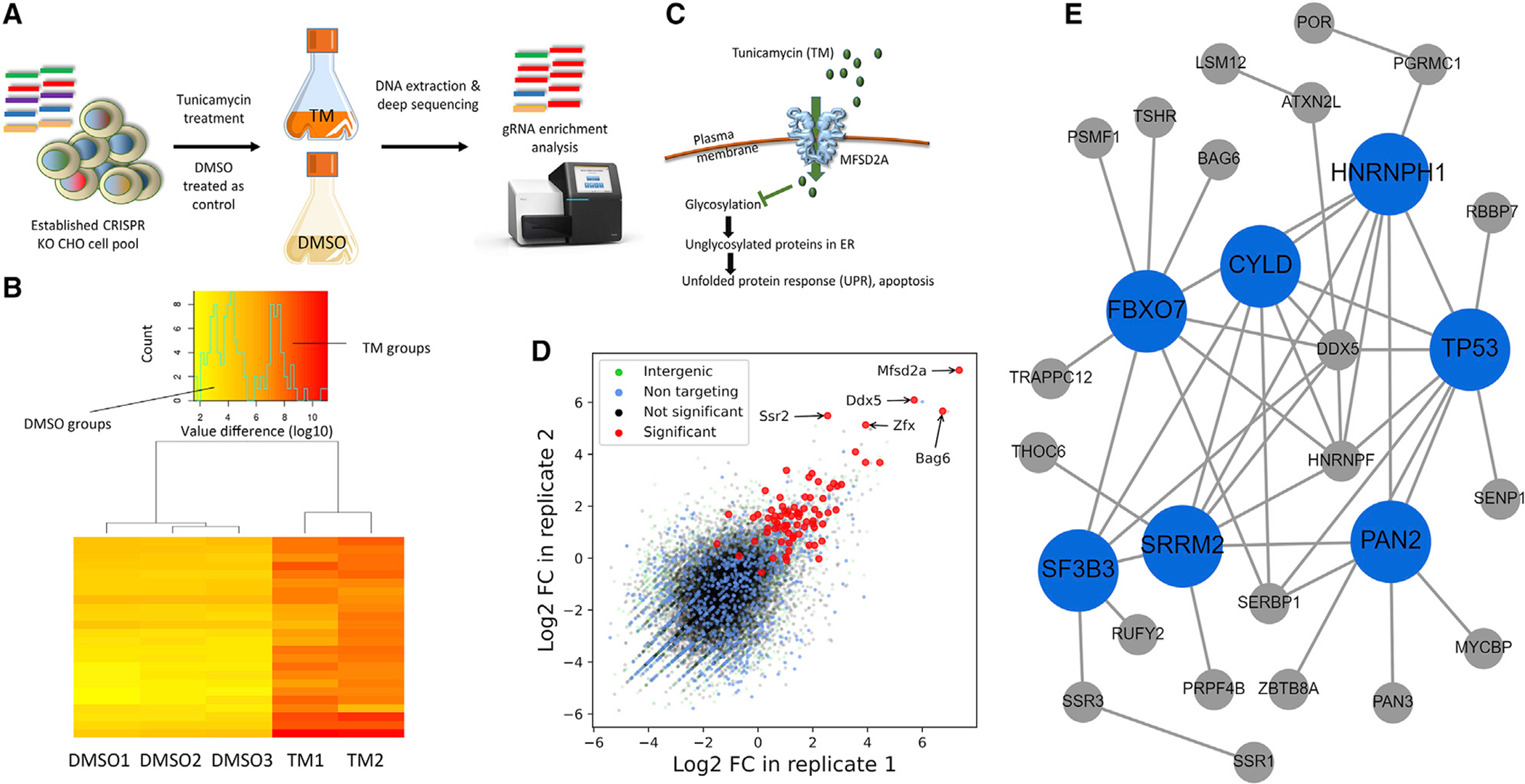
VF CRISPR screen for tunicamycin (TM)-resistant genes in CHO cells (A) Illustration of screen. Apoptosis in CHO cells was induced by TM treatment (20 ng/mL), and cells treated with DMSO (0.2%) were used as controls. (B) Heatmap of gRNA enrichment difference in TM-treated duplicates and control triplicates. This cluster analysis is based on the most abundant/depleted gRNAs. Log10 normalized read counts are color coded from lowest (yellow) to highest (red). (C) Major facilitator domain containing 2A (MFSD2A) transporter as a key mediator in the response to TM. (D) Fold change in gRNAs in VF CRISPR screen after TM treatment. Fold changes in the gRNAs targeting *Bag6*, and *Zfx* and the gRNAs targeting the positive control *Mfsd2a* are highlighted in red. (E) Network-topology-based analysis based on the protein-protein interactions of candidate genes whose KO would provide TM resistance in CHO cells.

The CRISPR KO CHO cell pool (60 mL, starting density 7 × 10^5^ cells/mL) was treated with TM (20 ng/mL) (Figure 5A). After 4 days of TM treatment, cells recovered for 5 days by being cultured in the medium without selection chemicals (Figure S4). The fold change in gRNAs in TM-treated and control cells was evaluated by NGS and computational analysis (Spahn et al., 2017), and the different fold-change patterns were observed in TM-treated and control groups (Figure 5B). We hypothesized that *Mfsd2a* (major facilitator domain containing 2A) would be a positive control, given that MFSD2A has been identified as an essential plasma membrane transporter of TM in a human cell line (Reiling et al., 2011) (Figure 5C). As expected, the gRNAs targeting *Mfsd2a* were significantly enriched after TM treatment (Figure 5D). In total, gRNAs targeting 77 genes enriched in this screen (Figure 5D, combined p < 0.01 in Table S6). Network topology-based analysis (Liao et al., 2019) demonstrated that 36 of these candidate genes showed a high degree of protein-protein interactions (PPIs) between candidate genes (Figure 5E). Further analysis of PPIs by using the STRING database (Szklarczyk et al., 2019) demonstrated that these candidates were significantly clustered (p = 7.42 × 10^−8^; Figure S5).

We compared our VF screen results with those obtained by using the smaller lentiviral library. Here the overlap was less impressive than for the proliferation screen. When looking only at genes targeted by both libraries, 11 genes were found to be significant in the lentiviral library and 11 in the VF library, but only 1 overlapping gene, *Degs1*, was identified.

### Validation of candidate genes

**Figure 6.**
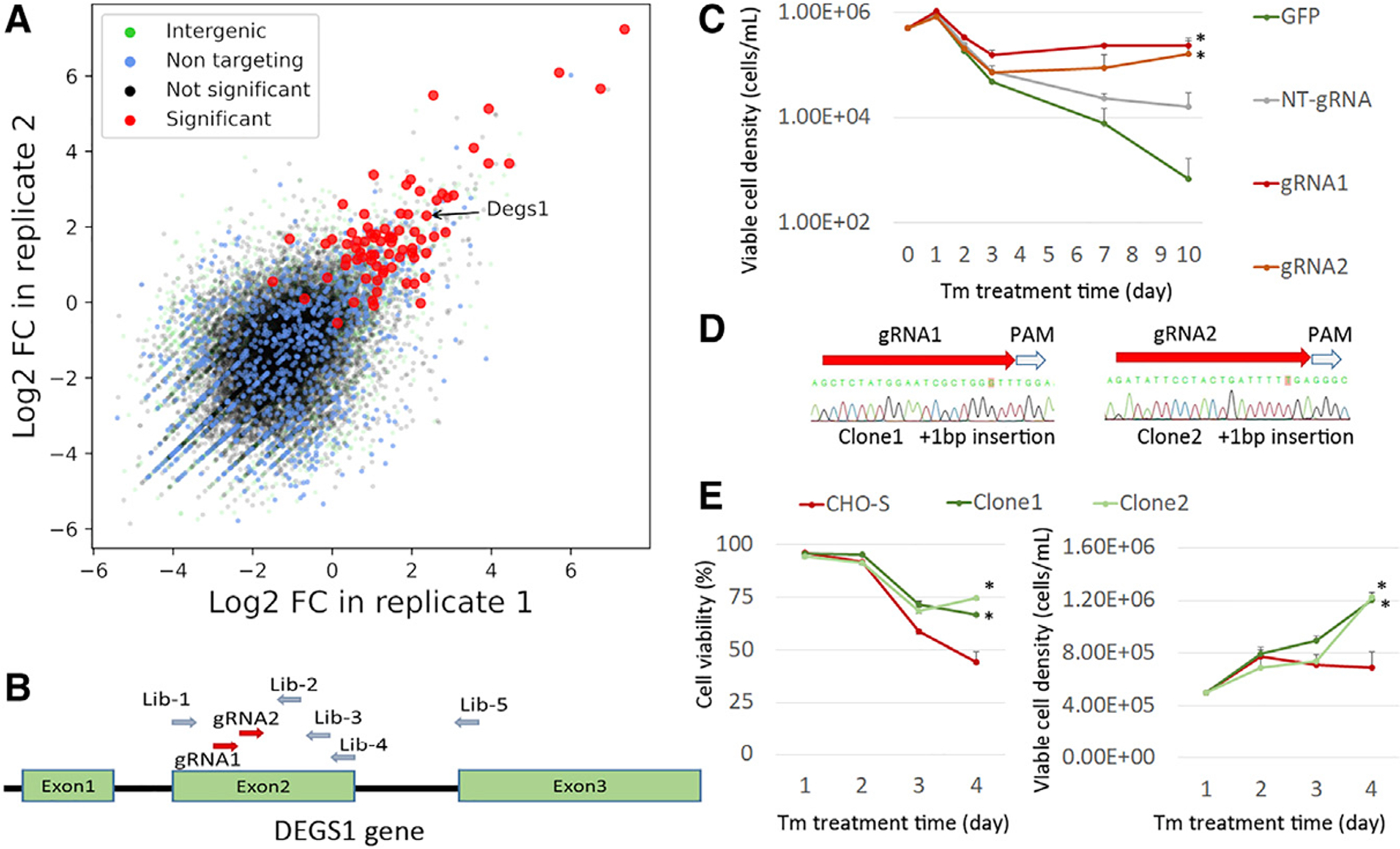
Validation of *Degs1* as candidate gene for KO to obtain ER-stress resistance (A) Fold change in gRNAs targeting *Degs1* in two replicates of VF CRISPR screen after TM treatment. (B) Two gRNAs were designed to target exon 2 of *DEGS1* in addition to the five gRNAs designed in the VF gRNA library. (C) Effects of transfection of two gRNAs in Cas9-CHO cells (continuously expressing Cas9) to obtain TM resistance. *p < 0.05. Statistical significance at day 10. (D and E) (D) Establishment of two cell lines with *DEGS1* KO and (E) their resistance to TM treatment. *p < 0.05. Statistical significance at day 4.

Degs1 (Delta 4-Desaturase, Sphingolipid 1) catalyzes the final step in the *de novo* biosynthesis of ceramides, and KO has been previously demonstrated to have an antiapoptotic effect (Breen et al., 2013; Siddique et al., 2013; Rodriguez-Cuenca et al., 2015; Zhu et al., 2016). In the screens we performed, gRNAs targeting Degs1 were enriched after ER stress in both the VF screen and the lentivirus screen (Figure 6A). To confirm the effects caused by Degs1 KO, we designed two additional gRNAs, also targeting Degs1, in addition to the five original gRNAs in the VF library (Figure 6B). Cas9-CHO cells with stable Cas9 expression were transfected with these two gRNAs, independently. Seven days after transfection, we treated the cell pools with TM (20 ng/mL) for an additional 7 days, and the cell density in each group was measured (Figure 6C). The cell growth curves indicated that the Cas9-CHO cells transfected with either gRNA targeting Degs1 survived better than the cells in the control groups. We further established two cell lines with isogenic Degs1 KO by co-transfection of Cas9 and gRNA-Degs1 in wild-type CHO-S cells followed by single-cell sorting and PCR detection and confirmed by Sanger sequencing (Figure 6D). Two clones were banked with +1 bp insertion near the gRNA targeting sequence (clone 1 was generated by gRNA1 and clone 2 was generated by gRNA2), and both of these Degs1 KO CHO cell lines demonstrated resistance to TM-induced apoptosis compared with wild-type CHO-S cells (Figure 6E).

**Figure 7.**
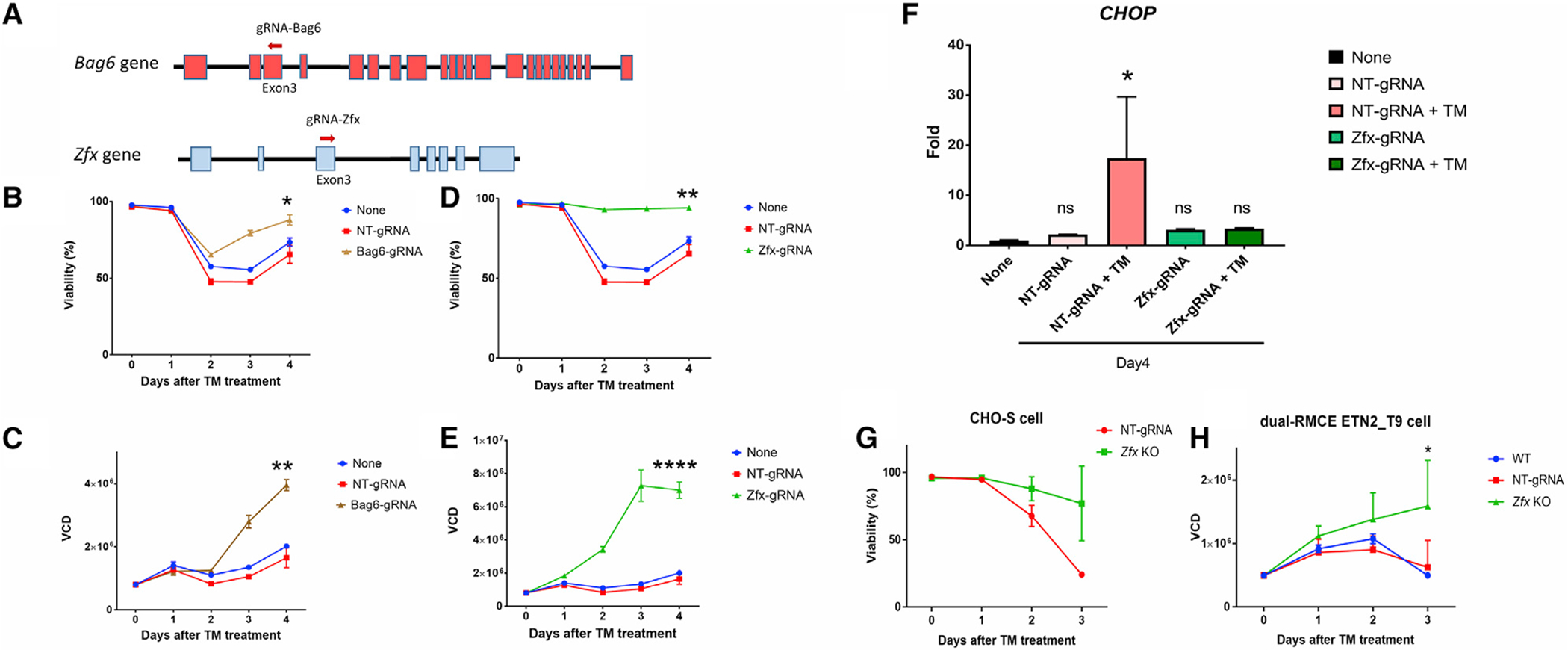
Validation of candidate genes for KO to obtain TM resistance (A–E) Shown in (A), one new gRNA for each gene was designed to target *Bag6* and *Zfx*. Effects of gRNA transfection in Cas9-CHO cells (constitutively expressing Cas9) on (B and D) cell viability and (C and E) viable cell density (VCD) targeting (B and C) *Bag6* and (D and E) *Zfx*. Seven days after transfection of gRNA, the transfected Cas9-CHO cells were treated with 20 ng/mL TM, and the viability and VCD were recorded for 4 days afterward. Statistical analysis of data at day 4 was performed (*p < 0.05, **p < 0.01, ****p < 0.0001 compared with “None” group). (F–H) As shown in (F), the ER stress marker *CHOP* expression was measured at day 4 after TM treatment in (D) and (E), or at day 4 after cell seeding in Figure S8. Effects of *Zfx* KO to obtain TM resistance in isogenic cell lines with *Zfx* KO using the parent cell line from (G) CHO-S cells (n = 3) or from (H) an Enbrel-producing cell line (dual-RMCE ETN2_T9, n = 2, *p < 0.05 compared with wild type [“WT”] group). The control cell lines were established by transfection with NT-gRNA and otherwise processed identical to the *Zfx* KO cell lines (n = 2 in [G] and [H]).

In addition, we selected *Bag6* (BCL2-Associated Athanogene) and *Zfx* (Zinc-Finger Protein X-Linked) for further validation, given that all the gRNAs targeting them significantly increased after TM treatment (Figure 5D). Two new gRNAs were designed targeting *Bag6* and *Zfx*, respectively (Figure 7A). Seven days after transfection of the new gRNA for each gene into CHO-Cas9 cells with stable Cas9 expression, the different cell pools were treated with TM (20 ng/mL) for an additional 4 days. Viability and viable cell density (VCD) improved for the CHO-Cas9 cells transfected with gRNAs targeting *Bag6* or *Zfx* compared with the control cells, which were transfected with NT-gRNA or GFP. For example, the *Bag6* mutant recovered 88% of viability by day 4 after TM treatment (Figure 7B), and the VCD doubled compared with the control groups (Figure 7C). Surprisingly, almost full resistance to TM was obtained in CHO cells after disruption of *Zfx*, wherein the cell viability after *Zfx* disruption remained above 90% (Figure 7D) and the VCD was almost 5 times more than that of the control groups 4 days after TM treatment (Figure 7E). Furthermore, the expression levels of the *CHOP* gene, a well-known ER stress marker, significantly increased after TM treatment in control cells but not in the cells with *Zfx* disruption (Figure 7F), indicating blockade of the ER stress pathway after *Zfx* KO. However, growth in TM-free culture medium showed that none these KOs gave an advantage to cell viability or VCD under normal conditions (Figure S6). We further investigated the effects of *Zfx* KO in isogenic CHO cell lines. We established three *Zfx* KO CHO-S cell lines by single-cell sorting and confirming the *Zfx* KO with Sanger sequencing. Resistance to TM was likewise observed in these three *Zfx* KO cell lines (Figure 7G). TM resistance obtained from *Zfx* KO was also observed in two established Enbrel-producing CHO cell lines (Sergeeva et al., 2020) (dual-RMCE ETN2_T9) with *Zfx* KO (Figure 7H). Thus, the VF CRISPR screen can successfully identify candidate genes via gRNA enrichment analysis.

## DISCUSSION

Here we present a new and improved VF CRISPR screen approach with low noise that works with standard CRISPR screen analysis tools. In our VF system, 92.5% of cells demonstrated single integration of the gRNA expression cassette, thus decreasing the biases in screens caused by no or multiple integrations of gRNA (Figure 1E). Targeted integration also decreases clone-to-clone variance on productivity compared with random insertion (Lee et al., 2015). Similarly, we see in our VF platform significantly reduced clonal variation in cell proliferation compared with the random insertions in lentiviral methods (Figure 1D). The gRNA coverage in our cell-based genome-wide gRNA library was 99.95% (Figure 1G). This is comparable to that achieved by a lentiviral strategy. Almost no gRNA expression cassettes were lost during the long-term cell culture (Figures 1F and 1G), indicating the stability of our platform. Therefore, our VF strategy improves on the genetic instability seen in lentiviral-transduced cells (Schambach et al., 2013; Rothe et al., 2014). In addition, the time cost for establishing the VF CRISPR screen platform is similar to that for establishing a lentiviral CRISPR screen platform (Figure S7).

Our platform also solved the problem that gRNA donor-integrated cells in the pool cannot be enriched by resistance-gene selection when random integration occurs. By applying Bxb1 RMCE to precisely integrate a donor-DNA-containing gRNA library with the promoter-less selection marker, the integrated cells could be enriched by puromycin selection in the final population, in which 99.78% of cells demonstrated successful integration of the gRNA expression cassette (Figure 1C). This efficiency is much higher than that achieved previously by a VF CRISPR screen approach based on homologous recombination, which showed an efficiency of around 30% (Rajagopal et al., 2016).

For the application of the VF CRISPR screen in our study, *Bag6* and *Zfx* are suggested for KO to reduce TM-induced apoptosis. Bag6 has previously been demonstrated to mediate apoptosis induced by ER stress (Desmots et al., 2008) and DNA damage (Sasaki et al., 2007; Krenciute et al., 2013). Similarly, a former study showed that Bag6-null mouse cells are resistant to cell death induced by thapsigargin, which is another chemical that induces ER stress (Desmots et al., 2008). Our study finds for the first time that *Zfx* KO provides absolute resistance to TM-induced apoptosis in CHO cells (Figure 7). It has previously been reported that TM treatment reduces the N-glycosylation of Zfx and that the non-glycosylated form of Zfx accumulates in the cell, possibly being the cause of ER stress and/or apoptosis (Zhu et al., 2013). That hypothesis would explain why *Zfx* KO provides resistance to TM treatment. However, several previous studies showed that downregulating the *Zfx* gene generally results in increased apoptosis in human A549 cells (Zha et al., 2013), malignant glioma cells (Zhou et al., 2011), B lymphocytes (Arenzana et al., 2009), and mouse embryonic stem cells and hematopoietic stem cells (Cellot and Sauvageau, 2007; Galan-Caridad et al., 2007), suggesting an incomplete understanding of the role of Zfx and the toxicity of unglycosylated Zfx.

In summary, this work establishes a genome-wide VF CRISPR screening strategy that we believe will be of interest in cases in which working with viruses for whatever reason is unfeasible. In addition, for CHO cells, we present an expressed genome-wide library that can be used for depletion or enrichment screening of phenotypes of interest in pharmaceutical protein production. Although applied here to CHO cells as an example, this approach can also be applied to other cell-based models. Thus, this approach presents a valuable method for precise VF genome-wide screens.

### Limitations of the study

The presented method does have some limitations. For one, it requires the host cell to be pre-engineered with a landing pad for the recombinase system to work. This makes it more difficult to work with primary cells and likewise cells that are difficult to transfect. One can also speculate that picking one specific genomic integration spot for the gRNA might specifically perturb phenotypes that are somehow affected by that particular genome location.

## STAR★METHODS

### RESOURCE AVAILABILITY

#### Lead contact

Further information and requests for resources and reagents should be directed to and will be fulfilled by the Lead Contact, Lasse Ebdrup Pedersen (laeb@dtu.dk).

#### Materials availability

All unique/stable reagents generated in this study are available from the Lead Contact with a completed Materials Transfer Agreement.

#### Data and code availability

Sequencing data and analysis scripts are available at Zenodo https://doi.org/10.5281/zenodo.4494299.

Sequencing data is also deposited in the SRA at NCBI, bioproject accession PRJNA699155.

Remaining data is available in the supplemental information.

### EXPERIMENTAL MODEL AND SUBJECT DETAILS

#### Cell culture and transfection

CHO-S cells (Thermo Fisher Scientific) were cultured in CD CHO medium supplemented with 8 mM L-Glutamine (Thermo Fisher Scientific) and 2 μL/mL Anti-Clumping reagent (AC, Life Technologies). Cells were maintained in Erlenmeyer shake flasks (Corning Inc., Acton, MA), incubated in a humidified incubator at 37°C, 5% CO2 at 120 rpm and passaged every 2–3 days. Viable cell density (VCD) and viability were monitored using the NucleoCounter NC-200 Cell Counter (ChemoMetec, Denmark). One day before transfection, cells were seeded into the culture medium without AC. On the day of transfection, cells were transfected using 3.75 μg of DNA packaged with 3.75 μL of FreeStyle Max transfection reagent (Thermo Fisher Scientific) per well of a 6-well plate (BD Biosciences) in 3 mL culture medium without AC and with 1E6 cells/mL. Specifically, for VF gRNA library transfection, 150 μg Bxb1 recombinase plasmid and 450 μg gRNA library donor were transfected into 480 mL CHO-attp-mCherry cells at the density of 1E6 cells/mL. The cells were divided into several 6-well plates and one day after transfection, the cells were combined into two 500 mL flasks. Transfection efficiencies were monitored using the transfection reagent included pmax-GFP plasmid and was generally observed to be between 60–90%.

### METHOD DETAILS

#### Expressed genes filtering and VF gRNA library design

RNA-seq data from 1558 CHO cell samples were analyzed to identify all expressed CHO genes from a recent genome assembly and annotation (Rupp et al., 2018). 18238 genes were expressed at TPM >1 in at least 5% of the samples; 27 genes were expressed between 0.5–1 TPM in at least 40% samples; 88 genes were expressed at <0.5 TPM in 85% samples. gRNAs were designed by DESKTOP Genetics based on the CH genome (Rupp et al., 2018) and proprietary Pacific Biosciences genome sequence data from CHO-S. This resulted in a library containing 75488 unique gRNAs of which 72149 target 15028 genes, 2218 target intergenic regions, 1051 are non-targeting gRNAs and finally 70 gRNA are targeting multiple genes (Table S1). gRNAs oligos were synthesized by Twist Biosciences (San Francisco, CA). The annealing oligos were cloned into the attB-Esp3I vector (See plasmid map and sequence) to make an attB-gRNA vector as donor DNA for RMCE following a protocol described previously (Joung et al., 2017).

#### Lentiviral library generation

The lentiviral cell library was made in a previous study (Karottki et al., 2021) where wild type CHO-S cells (Thermo Fisher Scientific) were transduced with the lentiviral library. Briefly, gRNAs involved in CHO cell metabolism were designed, synthesized, and packaged into lentivirus. The lentiviral library was used to transduce CHO-S cells at a low virus to cell ratio (MOI=0.25). The transduced cells were subject to puromycin selection to remove cells that did not receive a gRNA cassette. In order to facilitate comparison with our viral free library, we re-annotated these gRNAs. The lentiviral library consists of 16985 unique gRNAs of which 16744 target 2909 genes, 145 target intergenic regions, 89 are non-targeting gRNA and 7 gRNAs are targeting multiple genes (Table S2).

#### CHO-attp-mCherry cell line generation with RMCE landing pad and the RMCE of gRNA expression cassette

The CHO-attp-mCherry cell line was made CRISPR-mediated homology directed targeted integration of a RMCE landing pad into T2 genomic site in CHO-S cells (Thermo Fisher Scientific) as previously described (Lee et al., 2015; Petersen et al., 2018; Pristovšek et al., 2019). This cell line contains an mCherry coding sequence flanked by an attp sequence at the 5’ end and a mutant attp sequence at the 3’ end (pEF1a-attp-mCherry-HygroR-attp, landing pad vector is shown in plasmid map and sequence), and the 5’ and 3’ homology arms target at a transcriptionally active site within a non-coding region (T2 site). Vectors for RMCE were constructed by assembly of PCR fragments containing promoter-less puroR gene and gRNA cassette that was flanked by attB and mutant attB sequences. The cell with landing pad was transfected with RMCE gRNA plasmid (library) and Bxb1-recombinase vector in 3:1 ratio to exchange mCherry coding sequence with gRNA cassette. For Bxb1 recombinase expression, PSF-CMV-Bxb1 recombinase vector (see plasmid and sequence) was used. To maintain enough gRNA coverage (500 copies per gRNA), 480mL (1E6 viable cells/mL) of cells were transfected (while RMCE rate is measured as 4% in Figure S1). Three days after transfection, the transfected cell pool was selected with 10μg/mL puromycin. After 14–20 days, the cell pool was fully enriched to mCherry negative populations. The transfected cell pool was passaged every two days. After 9 and 25 days of gRNA RMCE, cells were analyzed by flow cytometry (BD FACSJazz cell sorter, BD Biosciences) to measure the mCherry density in the cell pool. The percentages of mCherry-negative cells on day 9 and day 25 were calculated to estimate the RMCE efficiency and confirm the cell-based gRNA library was completely established.

#### Copy number analysis using qPCR

Relative copy numbers of the integrated gRNA were determined by qPCR. Genomic DNA was extracted using the GeneJET Genomic DNA Purification Kit (Thermo Fisher Scientific) according to manufacturer instructions. qPCR was run on a QuantStudio 5 Real-Time PCR System (Agilent Technologies). Amplification was performed under the following conditions: 50°C for 2 min, 95°C for 10 min; 40x: 95°C for 15 s, 60°C for 1 min. Copy numbers of gRNA were determined with C1GALT1C1 as an internal control gene for normalization using SYBR Green qPCR kit (Thermo Fisher Scientific). Forward primer 5′-GCAGCCTTTCTATCTAGGACAC-3′ and reverse primer 5′-CCACCTTGTTCAGGACACTT-3′ are designed for C1GALT1C1 detection. Forward primer 5′-GCTTTATATATCTTGTGGAAAGGACGAAACACC-3′ and reverse primer 5′-CCGACTCGGTGCCACTTTTTCAA-3′ are designed for gRNA detection. Primer pairs were validated by melting curve analysis and primer efficiency test. A delta-delta threshold cycle (ΔΔCt) method was applied to calculate the copy number of gRNA integrated using previously single-copy calibrators. Each experiment was performed in technical triplicates using genomic DNA from CHO-S cells as a negative control.

#### Cas9-transfected cells enrichment

The cell-based gRNA library transfected with GFP was used as control. One day after transfection of Cas9-BSD vector (See plasmid map and sequence), the cells were treated with 10 μg/mL of blasticidin for 1 day and with 5 μg/mL of blasticidin for an additional one day. On day 4 after transfection the cells were cultured in medium without blasticidin for recovery. After day 8, the recovered cell pool was ready to be used for experiments. Cells in the control group were re-seeded to 1E6 cells/mL whenever the viable cell density reached 5E6 cells/mL.

#### Preparation for NGS

50 μL PCR reactions with 2.5–3 μg input gDNA per reaction were run using Phusion®Hot Start II High-Fidelity DNA Polymerase (Thermo Fisher Scientific) (95°C for 3 min; 30 times: 98°C for 45s, 60°C for 30 s, 72°C for 1 min; 72°C for 7 min) using primers identical in the method of copy number analysis. PCR products were purified by Macherey-Nagel NucleoSpin Gel & PCR purification kit. The amplicons were added with Illumina NGS index with adapters using DNA ligation. The resulting library was quantified with Qubit using the dsDNA HS Assay Kit (Thermo Fisher Scientific) and the fragment size was determined using a 2100 Bioanalyzer Instrument (Agilent) before running the samples on a NextSeq 500 sequencer (Illumina).

#### Clone variance analysis

Single cells were sorted from the LV-lib pool (before Cas9 transfection) and VF library pool (CHO-S cells infected with lentiviral gRNA library but without Cas9) and deposited into a 96-well plate by BD FACSJazz cell sorter (BD Biosciences). 14 days after single cell sorting, the confluence of the emerging colonies in each well was measured by Celigo Cell Imaging Cytometer (Nexcelom Bioscience). Colonies with confluence lower than 10% were filtered out. The clones of 4 plates from VF library and 4 plates from LV-lib was calculated.

#### Gene disruption validation

One new gRNA for each gene was designed to target Bag6 and *Zfx* gene, respectively. The target sequences of gRNAs targeting Bag6, *Zfx* are GGCATTCCGGTCATGAACAGAGG (Bag6-gRNA) and TTCTTCTGAAACAACATCGGCGG (Zfx-gRNA), respectively. Detailed materials and methods for gRNA design and the gRNA vector construction are elaborated in our previous study (Xiong et al., 2019). The CHO-Cas9 cells with stable expression of Cas9 was transfected with gRNAs. 7 days after transfection, the recovered cells were cultured in the medium described as above with 20ng/mL tunicamycin (TM) for 4 days as indicated in legends. The CHO-Cas9 cells transfected with GFP or NT-gRNA were treated with 20ng/mL TM as control. The medium was never changed during these 4 days of TM treatment. The cell viability and the VCD were measured using the NucleoCounter NC-200 Cell Counter (ChemoMetec, Denmark) during this TM treatment process. A non-targeting gRNA was used as control as described previously (Karottki et al., 2020).

#### Generation of CHO-Cas9 cell line and CHO-attP-mCherry/Cas9 cell line

To establish a cell platform for stably expressing Cas9, a CHO-Cas9 cell line was generated from a parent cell line containing a RMCE landing pad. The parent cell line was made by CRISPR-mediated homology directed targeted integration of CHO-S cells (Life Technologies) as previously described (Lee et al., 2015; Petersen et al., 2018; Pristovšek et al., 2019). The parent cell line contains a mCherry coding sequence flanked by a loxP sequence at the 5’ end and a lox2272 sequence at the 3’ end (pEF1-loxP-mCherry- lox2272-BGHpA), and the 5’ and 3’ homology arms target a transcriptionally active site within a non-coding region (See plasmid map and sequence). Promoter-less RMCE vectors were constructed by assembly of PCR fragments containing the Cas9 region flanked by loxP and lox2272 sequences. The parent cell line was transfected with RMCE Cas9 donor plasmids (See plasmid map and sequence) and Cre-recombinase vector in 3:1 ratio to exchange the mCherry coding sequence with Cas9. For Cre recombinase expression, PSF-CMV-CRE recombinase vector (OGS591, Sigma-Aldrich) was used. Transfected cell pool was passaged twice after transfection. After 7 days, mCherry-negative single cell sorting was performed by FACS using a BD FACSJazz cell sorter (BD Biosciences). After 14 days, the monoclonal CHO-Cas9 cell line was established after verification of proper integration and confirmation of single copy of Cas9. The methods and the materials for generating this CHO-Cas9 cell line were described before in our study (Hart et al., 2015). By using CHO-Cas9 cell line as the parent cell line, attP RMCE landing pad was inserted into the T2 site as the protocol described above to generate CHO-attP-mCherry/Cas9 cell line.

#### gRNA design for *Mgat1* KO and *Mgat1* editing analysis

A gRNA targeting *Mgat1* (gRNA-Mgat1) was designed to target GTGGAGTTGGAGCGGCAGCGGGG. gRNA-Mgat1 was cloned into the attB vector to achieve RMCE of gRNA-Mgat1 showed in Figures 1H and S2. gRNA-Mgat1 was cloned into identical gRNA vector as described in our previous study for *Mgat1* KO by transient expression in Figure 1H. A pair of primers with the sequence 5′-TTCTGGACACGCCCAGC-3′ (forward) and 5′-GCCACGGTGGGCACTTT-3′ was applied to detect *Mgat1* editing by Sanger sequencing (Figure S2) or by NGS analysis (Figure1H). A non-targeting gRNA was used as control for confirming the *Mgat1* KO (Figure S2).

#### Establishment of *Zfx* KO cell lines

CHO-S WT cells or dual-RMCE ETN2_T9 CHO cells were transfected with Zfx-gRNA described above. The pools were single cell sorted by FACS into 96-well cell culture plates. After 14 days, the single cell derived populations were tested for double KO of Zfx using sanger sequencing of amplicons generated with the following primers: forward: 5′- TACATGTGGCTGACGTTGGT-3′ and reverse: 5′- GGAAATCATAAGGTAGTCCTCACA-3′. CHO-S WT cells and dual-RMCE ETN2_T9 cells transfected with NT-gRNA and otherwise processed identically as above were used as control (Figures 7G and 7H).

#### Plasmid maps and sequences

Sequences and maps of all plasmids can be found in the zenodo data repository https://doi.org/10.5281/zenodo.4494299

### QUANTIFICATION AND STATISTICAL ANALYSIS

#### NGS data analysis

The gRNA coverage is calculated by the percentage of gRNA with at least 1 read in the library and skew ratio analysis was calculated by the ratio of reads at cumulative percentage 90% to 10%, following suggestions in former CRISPR GeCKO library screens protocol from Feng Zhang’s lab (Konermann et al., 2015; Joung et al., 2017). For gRNA fold change analysis, raw FASTQ files were analyzed using default parameters in PinAPL-PY (Spahn et al., 2017) (http://pinapl-py.ucsd.edu/) along with a file containing the sequences for all gRNAs contained in the library. Top candidates for enriched and for depleted gRNAs were ranked by the adjusted robust rank aggregation (aRRA) method (Li et al., 2014) and filtered for significance, compared between the replicates and used for verification of the screen. A gene was labelled “significant” if it was both statistically significant on the gene levels as determined by pinapl-py and statistically significant on the gRNA level for at least 3 gRNAs and at least half the gRNAs targeting the gene. Biological process depletion was performed in the PANTHER classification system (Mi et al., 2019a, 2019b) (http://www.pantherdb.org). The list of gRNA fold change was tested against the PANTHER GO-Slim Biological Process annotation set. Network topology-based analysis based on protein-protein-interaction (PPI) of candidate genes was performed in WEB-based GEne SeT AnaLysis Toolkit (Wang et al., 2017a; Liao et al., 2019). Network Retrieval & Prioritization method was applied in this NTA analysis (http://www.webgestalt.org). The PPI analysis was confirmed in STRING database (string-db.org) (Szklarczyk et al., 2019). Essential genes in human and mouse were obtained from the DEG database (Luo et al., 2014) for essential genes (http://tubic.tju.edu.cn/deg). Essential gene lists of K562 cell and Hela cell were obtained from previous CRISPR screen studies (Hart et al., 2015; Wang et al., 2015) collected in the essential gene database (http://www.essentialgene.org/).

Raw data, PinAPL-PY output, and data analysis scripts can be downloaded from https://doi.org/10.5281/zenodo.4494299

Details of statistical tests can be found in relevant figure legends.

## Supplementary Material

Table S6. Significant genes TM screen, related to Figure 5

Table S5. Overlapping genes, related to Figure 4

Table S3. GO analysis of depleted gRNAs, related to Figure 4

Table S4. Essential gene screen, related to Figure 4

Table S2. LV gRNA library design, related to Figure 1

Document S1. Figures S1–S7

Table S1. VF gRNA library design, related to Figure 1

## Figures and Tables

**Table:** KEY RESOURCES TABLE

REAGENT or RESOURCE	SOURCE	IDENTIFIER
Deposited data		
Raw and analyzed data	This paper	https://doi.org/10.5281/zenodo.4494299
Raw NGS in NCBI SRA (also available in the zenodo repo)	This paper	NCBI bioproject accession PRJNA699155
Experimental models: Cell lines		
CHO-S	Thermo Fisher Scientific	Cat R80007
Oligonucleotides		
All oligos can be found in the zenodo repo	This paper	https://doi.org/10.5281/zenodo.4494299
Recombinant DNA		
All plasmids can be found in the zenodo repo	This paper	https://doi.org/10.5281/zenodo.4494299
Software and algorithms		
Pinapl-py	Spahn et al., 2017	http://pinapl-py.ucsd.edu
WEB-based GEne SeT AnaLysis Toolkit	Wang et al., 2017a; Liao et al., 2019	http://www.webgestalt.org/
STRING-DB	Szklarczyk et al., 2019	https://string-db.org
Panther-db	Mi et al., 2019a, 2019b	http://www.pantherdb.org/

## Highlights

- A recombinase-based method for virus-free, genome-wide CRISPR knockout screens
- Utilizes the same libraries and analysis tools as traditional virus-based screens
- Avoids noise associated with virus-based random insertions into the genome

## MOTIVATION

Although lentivirus-based delivery of genome-wide CRISPR screen components has proven successful, there are situations in, e.g., industry and hospitals where working with live viruses is difficult or simply not an option. For those situations we have developed an alternative to virus-based, genome-wide CRISPR screens that retains compatibility with the software tools developed for analyzing the results, takes a similar amount of time, and offers improved signal-to-noise ratio.

## References

[R1] Adamson B, Norman TM, Jost M, Cho MY, Nuñez JK, Chen Y, Villalta JE, Gilbert LA, Horlbeck MA, Hein MY, (2016). A multiplexed single-cell CRISPR screening platform enables systematic dissection of the unfolded protein response. Cell 167, 1867–1882.e21. 10.1016/j.cell.2016.11.048. 27984733 PMC5315571

[R2] Arenzana TL, Smith-Raska MR, and Reizis B (2009). Transcription factor Zfx controls BCR-induced proliferation and survival of B lymphocytes. Blood 113, 5857–5867. 10.1182/blood-2008-11-188888. 19329779 PMC2700322

[R3] Borth N, Mattanovich D, Kunert R, and Katinger H (2008). Effect of increased expression of protein disulfide isomerase and heavy chain binding protein on antibody secretion in a recombinant CHO cell line. Biotechnol. Prog 21, 106–111. 10.1021/bp0498241. 15903247

[R4] Breen P, Joseph N, Thompson K, Kraveka JM, Gudz TI, Li L, Rahmaniyan M, Bielawski J, Pierce JS, Van Buren E, (2013). Dihydroceramide desaturase knockdown impacts sphingolipids and apoptosis after photodamage in human head and neck squamous carcinoma cells. Anticancer Res. 33, 77–84. 23267130 PMC3905466

[R5] Cellot S, and Sauvageau G (2007). Zfx: at the crossroads of survival and self-renewal. Cell 129, 239–241. 10.1016/j.cell.2007.04.002. 17448983

[R6] Desmots F, Russell HR, Michel D, and McKinnon PJ (2008). Scythe regulates apoptosis-inducing factor stability during endoplasmic reticulum stress-induced apoptosis. J. Biol. Chem 283, 3264–3271. 10.1074/jbc.M706419200. 18056262

[R7] Galan-Caridad JM, Harel S, Arenzana TL, Hou ZE, Doetsch FK, Mirny LA, and Reizis B (2007). Zfx controls the self-renewal of embryonic and hematopoietic stem cells. Cell 129, 345–357. 10.1016/j.cell.2007.03.014. 17448993 PMC1899089

[R8] Hart T, Chandrashekhar M, Aregger M, Steinhart Z, Brown KR, MacLeod G, Mis M, Zimmermann M, Fradet-Turcotte A, Sun S, (2015). High-resolution CRISPR screens reveal fitness genes and genotype-specific cancer liabilities. Cell 163, 1515–1526. 10.1016/j.cell.2015.11.015. 26627737

[R9] Joung J, Konermann S, Gootenberg JS, Abudayyeh OO, Platt RJ, Brigham MD, Sanjana NE, and Zhang F (2017). Genome-scale CRISPR-Cas9 knockout and transcriptional activation screening. Nat. Protoc 12, 828–863. 10.1038/nprot.2017.016. 28333914 PMC5526071

[R10] Karottki K.J.la C., Hefzi H, Li S, Pedersen LE, Spahn PN, Joshi C, Ruckerbauer D, Bort JAH, Thomas A, Lee JS, (2021). A metabolic CRISPR-Cas9 screen in Chinese hamster ovary cells identifies glutamine-sensitive genes. Metab. Eng 66, 114–122. 10.1016/j.ymben.2021.03.017. 33813034 PMC8193919

[R11] Karottki K.J.la C., Hefzi H, Xiong K, Shamie I, Hansen AH, Li S, Pedersen LE, Li S, Lee JS, Lee GM, (2020). Awakening dormant glycosyltransferases in CHO cells with CRISPRa. Biotechnol. Bioeng 117, 593–598. 10.1002/bit.27199. 31631317 PMC7261405

[R12] Kim HS, Lee K, Bae S, Park J, Lee C-K, Kim M, Kim E, Kim M, Kim S, Kim C, (2017). CRISPR/Cas9-mediated gene knockout screens and target identification via whole-genome sequencing uncover host genes required for picornavirus infection. J. Biol. Chem 292, 10664–10671. 10.1074/jbc.M117.782425. 28446605 PMC5481571

[R13] Konermann S, Brigham MD, Trevino AE, Joung J, Abudayyeh OO, Barcena C, Hsu PD, Habib N, Gootenberg JS, Nishimasu H, (2015). Genome-scale transcriptional activation by an engineered CRISPR-Cas9 complex. Nature 517, 583–588. 10.1038/nature14136. 25494202 PMC4420636

[R14] Krenciute G, Liu S, Yucer N, Shi Y, Ortiz P, Liu Q, Kim B-J, Odejimi AO, Leng M, Qin J, (2013). Nuclear BAG6-UBL4A-GET4 complex mediates DNA damage signaling and cell death. J. Biol. Chem 288, 20547–20557. 10.1074/jbc.M112.443416. 23723067 PMC3711319

[R15] Le Fourn V, Girod P-A, Buceta M, Regamey A, and Mermod N (2014). CHO cell engineering to prevent polypeptide aggregation and improve therapeutic protein secretion. Metab. Eng 21, 91–102. 10.1016/j.ymben.2012.12.003. 23380542

[R16] Lee JS, Kallehauge TB, Pedersen LE, and Kildegaard HF (2015). Site-specific integration in CHO cells mediated by CRISPR/Cas9 and homology-directed DNA repair pathway. Sci. Rep 5, 8572. 10.1038/srep08572. 25712033 PMC4339809

[R17] Li W, Xu H, Xiao T, Cong L, Love MI, Zhang F, Irizarry RA, Liu JS, Brown M, and Liu XS (2014). MAGeCK enables robust identification of essential genes from genome-scale CRISPR/Cas9 knockout screens. Genome Biol. 15, 554. 10.1186/s13059-014-0554-4. 25476604 PMC4290824

[R18] Liao Y, Wang J, Jaihnig EJ, Shi Z, and Zhang B (2019). WebGestalt 2019: gene set analysis toolkit with revamped UIs and APIs. Nucleic Acids Res. 47, W199–W205. 10.1093/nar/gkz401. 31114916 PMC6602449

[R19] Liu Y, Cao Z, Wang Y, Guo Y, Xu P, Yuan P, Liu Z, He Y, and Wei W (2018). Genome-wide screening for functional long noncoding RNAs in human cells by Cas9 targeting of splice sites. Nat. Biotechnol 36, 1203–1210. 10.1038/nbt.4283. 30395134

[R20] Luo H, Lin Y, Gao F, Zhang C-T, and Zhang R (2014). DEG 10, an update of the database of essential genes that includes both protein-coding genes and noncoding genomic elements: Table 1. Nucleic Acids Res. 42, D574–D580. 10.1093/nar/gkt1131. 24243843 PMC3965060

[R21] Mi H, Muruganujan A, Ebert D, Huang X, and Thomas PD (2019a). PANTHER version 14: more genomes, a new PANTHER GO-slim and improvements in enrichment analysis tools. Nucleic Acids Res. 47, D419–D426. 10.1093/nar/gky1038. 30407594 PMC6323939

[R22] Mi H, Muruganujan A, Huang X, Ebert D, Mills C, Guo X, and Thomas PD (2019b). Protocol Update for large-scale genome and gene function analysis with the PANTHER classification system (v.14.0). Nat. Protoc 14, 703–721. 10.1038/s41596-019-0128-8. 30804569 PMC6519457

[R23] Mohan C, and Lee GM (2010). Effect of inducible co-overexpression of protein disulfide isomerase and endoplasmic reticulum oxidoreductase on the specific antibody productivity of recombinant Chinese hamster ovary cells. Biotechnol. Bioeng 107, 337–346. 10.1002/bit.22781. 20506311

[R24] Petersen SD, Zhang J, Lee JS, Jakočiūnas T, Gray LM, Kildegaard HF, Keasling JD, and Jensen MK (2018). ‘Modular 5′-UTR hexamers for context-independent tuning of protein expression in eukaryotes’. Nucleic Acids Res. 10.1093/nar/gky734. PMC6265478 30124898

[R25] Prashad K, and Mehra S (2015). Dynamics of unfolded protein response in recombinant CHO cells. Cytotechnology 67, 237–254. 10.1007/s10616-013-9678-8. 24504562 PMC4329310

[R26] Pristovšek N, Nallapareddy S, Gray LM, Hefzi H, Lewis NE, Rugbjerg P, Hansen HG, Lee GM, Andersen MR, and Kildegaard HF (2019). Systematic evaluation of site-specific recombinant gene expression for programmable mammalian cell engineering. ACS Synth. Biol 8, 758–774. 10.1021/acssynbio.8b00453. 30807689

[R27] Rajagopal N, Srinivasan S, Kooshesh K, Guo Y, Edwards MD, Banerjee B, Syed T, Emons BJM, Gifford DK, and Sherwood RI (2016). High-throughput mapping of regulatory DNA. Nat. Biotechnol 34, 167–174. 10.1038/nbt.3468. 26807528 PMC5108523

[R28] Reiling JH, Clish CB, Carette JE, Varadarajan M, Brummelkamp TR, and Sabatini DM (2011). A haploid genetic screen identifies the major facilitator domain containing 2A (MFSD2A) transporter as a key mediator in the response to tunicamycin. Proc. Natl. Acad. Sci. U S A 108, 11756–11765. 10.1073/pnas.1018098108. 21677192 PMC3141996

[R29] Rodriguez-Cuenca S, Barbarroja N, and Vidal-Puig A (2015). Dihydroceramide desaturase 1, the gatekeeper of ceramide induced lipotoxicity. Biochim. Biophys. Acta 1851, 40–50. 10.1016/j.bbalip.2014.09.021. 25283058

[R30] Rothe M, Modlich U, and Schambach A (2014). Biosafety challenges for use of lentiviral vectors in gene therapy. Curr. Gene Ther 13, 453–468. 10.2174/15665232113136660006. 24195603

[R31] Rupp O, MacDonald ML, Li S, Dhiman H, Polson S, Griep S, Heffner K, Hernandez I, Brinkrolf K, Jadhav V, (2018). A reference genome of the Chinese hamster based on a hybrid assembly strategy. Biotechnol. Bioeng 115, 2087–2100. 10.1002/bit.26722. 29704459 PMC6045439

[R32] Sasaki T, Gan EC, Wakeham A, Kornbluth S, Mak TW, and Okada H (2007). HLA-B-associated transcript 3 (Bat3)/Scythe is essential for p300-mediated acetylation of p53’. Genes Dev. 21, 848–861. 10.1101/gad.1534107. 17403783 PMC1838535

[R33] Schambach A, Zychlinski D, Ehrnstroem B, and Baum C (2013). Biosafety features of lentiviral vectors. Hum. Gene Ther 24, 132–142. 10.1089/hum.2012.229. 23311447 PMC3581032

[R34] Sergeeva D, Lee GM, Nielsen LK, and Gray LM (2020). Multicopy targeted integration for accelerated development of high-producing Chinese hamster ovary cells. ACS Synth. Biol 9, 2546–2561. 10.1021/acssynbio.0c00322. 32835482

[R35] Siddique MM, Li Y, Wang L, Ching J, Mal M, Ilkayeva O, Wu YJ, Bay BH, and Summers SA (2013). Ablation of dihydroceramide desaturase 1, a therapeutic target for the treatment of metabolic diseases, simultaneously stimulates anabolic catabolic signaling. Mol. Cell Biol 33, 2353–2369. 10.1128/MCB.00226-13. 23547262 PMC3648062

[R36] Spahn PN, Bath T, Weiss RJ, Kim J, Esko JD, Lewis NE, and Harmendy O (2017). PinAPL-Py: a comprehensive web-application for the analysis of CRISPR/Cas9 screens. Sci. Rep 7, 15854. 10.1038/s41598-017-16193-9. 29158538 PMC5696473

[R37] Szklarczyk D, Gable AL, Lyon D, Junge A, Wyder S, Huerta-Cepas J, Simonovic M, Doncheva NT, Morris JH, Bork P, (2019). ‘STRING v11: protein–protein association networks with increased coverage, supporting functional discovery in genome-wide experimental datasets’. Nucleic Acids Res. 47, D607–D613. 10.1093/nar/gky1131. 30476243 PMC6323986

[R38] Tigges M, and Fussenegger M (2006). Xbp1-based engineering of secretory capacity enhances the productivity of Chinese hamster ovary cells. Metab. Eng 8, 264–272. 10.1016/j.ymben.2006.01.006. 16635796

[R39] Walsh G (2018). Biopharmaceutical benchmarks 2018. Nat. Biotechnol 36, 1136–1145. 10.1038/nbt.4305. 30520869

[R40] Wang J, Vasaikar S, Shi Z, Greer M, and Zhang B (2017a). WebGestalt 2017: a more comprehensive, powerful, flexible and interactive gene set enrichment analysis toolkit. Nucleic Acids Res. 45, W130–W137. 10.1093/nar/gkx356. 28472511 PMC5570149

[R41] Wang T, Yu H, Hughes NW, Liu B, Kendirli A, Klein K, Chen WW, Lander ES, and Sabatini DM (2017b). Gene essentiality profiling reveals gene networks and synthetic lethal interactions with oncogenic ras. Cell 168, 890–903.e15. 10.1016/j.cell.2017.01.013. 28162770 PMC5445660

[R42] Wang T, Birsoy K, Hughes NW, Krupczak KM, Post Y, Wei JJ, Lander ES, and Sabatini DM (2015). Identification and characterization of essential genes in the human genome. Science 350, 1096–1101. 10.1126/science.aac7041. 26472758 PMC4662922

[R43] Xiong K, Marquart KF, Cour Karottki KJ, Li S, Shamie I, Lee JS, Gerling S, Yeo NC, Chavez A, Lee GM, (2019). Reduced apoptosis in Chinese hamster ovary cells via optimized CRISPR interference. Biotechnol. Bioeng 116, 1813–1819. 10.1002/bit.26969. 30883679 PMC6545153

[R44] Zha W, Cao L, Shen Y, and Huang M (2013). Roles of Mir-144-ZFX Pathway in Growth Regulation of Non-Small-Cell Lung Cancer. PLoS One 8, e74175. 10.1371/journal.pone.0074175. 24066116 PMC3774613

[R45] Zhou Y, Su Z, Huang Y, Sun T, Chen S, Wu T, Chen G, Xie X, Li B, and Du Z (2011). The Zfx gene is expressed in human gliomas and is important in the proliferation and apoptosis of the human malignant glioma cell line U251. J. Exp. Clin. Cancer Res 30, 114. 10.1186/1756-9966-30-114. PMC3259083 22185393

[R46] Zhu Z, Li K, Xu D, Liu Y, Tang H, Xie Q, Xie L, Liu J, Wang H, Gong Y, (2013). ZFX regulates glioma cell proliferation and survival in vitro and in vivo. J. Neuro Oncol 112, 17–25. 10.1007/s11060-012-1032-z. 23322077

[R47] Zhu Q, Yang J, Zhu R, Jiang X, Li W, He S, and Jin J (2016). Dihydroceramide-desaturase-1-mediated caspase 9 activation through ceramide plays a pivotal role in palmitic acid-induced HepG2 cell apoptosis. Apoptosis 21, 1033–1044. 10.1007/s10495-016-1267-9. 27364952

