# An optimized genome-wide, virus-free CRISPR screen for mammalian cells

**DOI:** 10.1016/j.crmeth.2021.100115

**Published:** 2021-11-22

**Authors:** Kai Xiong, Karen Julie la Cour Karottki, Hooman Hefzi, Songyuan Li, Lise Marie Grav, Shangzhong Li, Philipp Spahn, Jae Seong Lee, Ildze Ventina, Gyun Min Lee, Nathan E. Lewis, Helene Faustrup Kildegaard, Lasse Ebdrup Pedersen

## Main text

(Cell Reports Methods *1*, 100062-1–100062-11; e1–e4, August 23, 2021)

As a result of an author oversight in the originally published version of this article, the author Helene Faustrup Kildegaard was omitted from the author list. The author’s name has now been added to the author list in the article online.

