# Supplementary material for: An optimized genome-wide, virus-free CRISPR screen for mammalian cells
Source: Cell Rep Methods. 2021 Aug 4;1(4):100062. doi: 10.1016/j.crmeth.2021.100062 (PMC8687118; doi:10.1016/j.crmeth.2021.100062)
Supplement: Document S1. Figures S1–S7 [file mmc1.pdf]

Cell Reports Methods, Volume *1*

Supplemental information

An optimized genome-wide, virus-free CRISPR screen for mammalian cells

Kai Xiong, Karen Julie la Cour Karottki, Hooman Hefzi, Songyuan Li, Lise Marie Grav, Shangzhong Li, Philipp Spahn, Jae Seong Lee, Ildze Ventina, Gyun Min Lee, Nathan E. Lewis, Helene Faustrup Kildegaard, and Lasse Ebdrup Pedersen

SUPPLEMENTARY FIGURES AND PLASMID MAPS

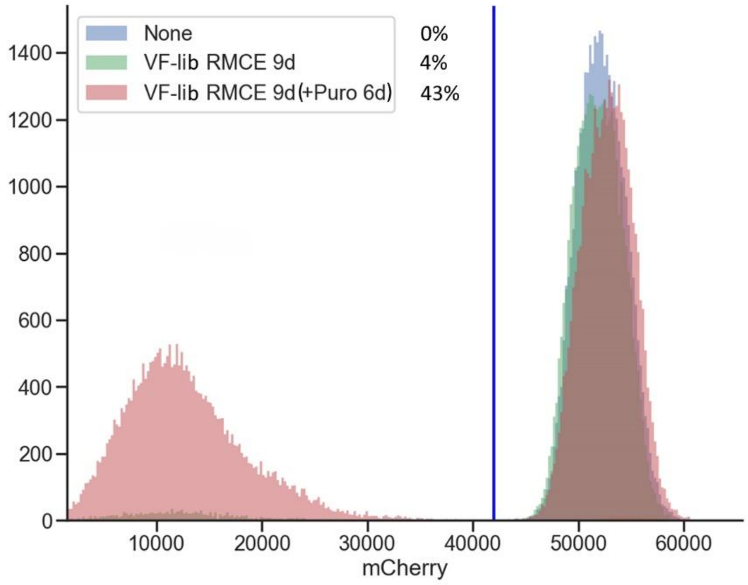

Figure S1 RMCE efficiency, related to STAR Methods "Cell culture and transfection"

mCherry density in CHO-attp-mCherry cell lines with or without RMCE of the VF gRNA library. CHO-attp-mCherry cell lines are transfected with the VF-gRNA library and Bxb1 recombinase. After 9 days of transfection, the mCherry density was measured by flow cytometry. The percentage of mCherry negative cells is shown here (mCherry gating = 42000). CHO-attp-mCherry cells without transfection were applied as a control.

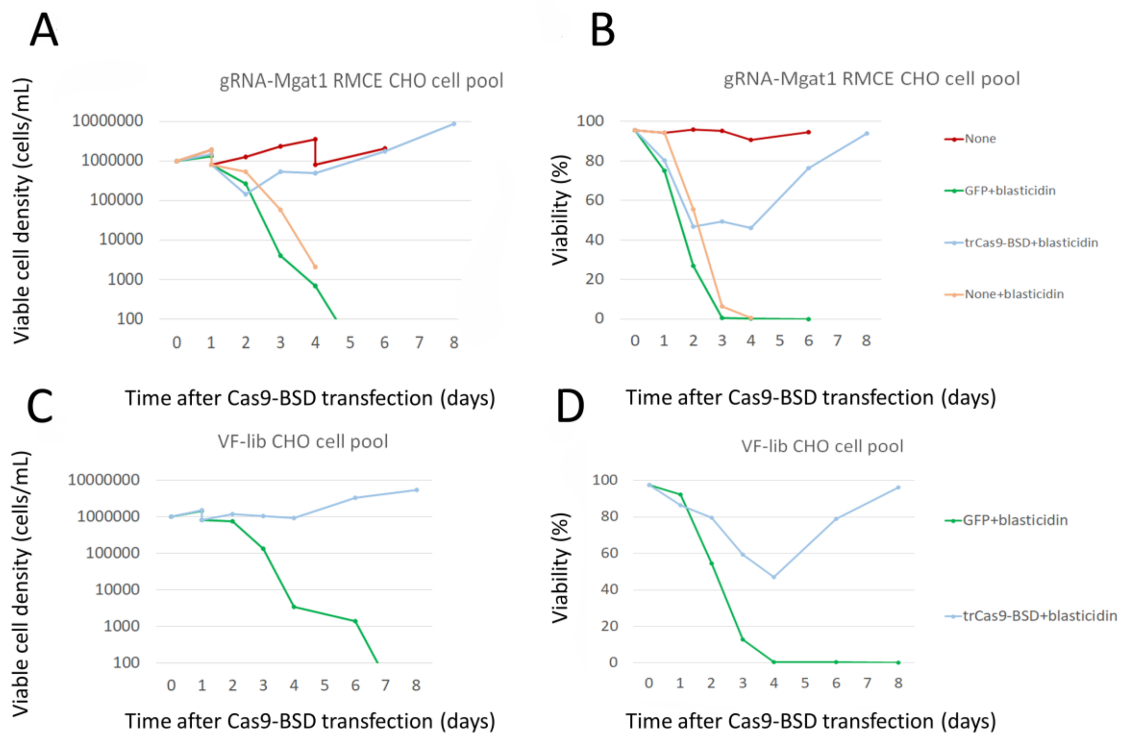

Figure S2 Effects of blasticidin selection on cell proliferation and viability, related to STAR Methods section “gRNA design for *Mgat1* KO and *Mgat1* editing analysis”

(**A**) Viable cell density and (**B**) viability of different treated cell pools with RMCE of gRNA-Mgat1 presented in **Figure 1h**. The identical cell pool transfected with GFP was used as a control. (**C**) Viable cell density and (**D**) viability of cell-based gRNA library during the Cas9-transfection enrichment. The cell-based gRNA library transfected with GFP was used as a control. 1 day after transfection of Cas9-BSD, the cells were treated with 10 µg/mL of blasticidin for 1 day and with 5 µg/mL of blasticidin for an additional day. On day 4 after transfection the cells were cultured in medium without blasticidin for recovery. After day 8 the recovered cell pool can be used for further experiments. Cells in the control group would be re-seeded to $1x10^6$ cells/mL if the viable cell density reached $5x10^6$ cells/mL.

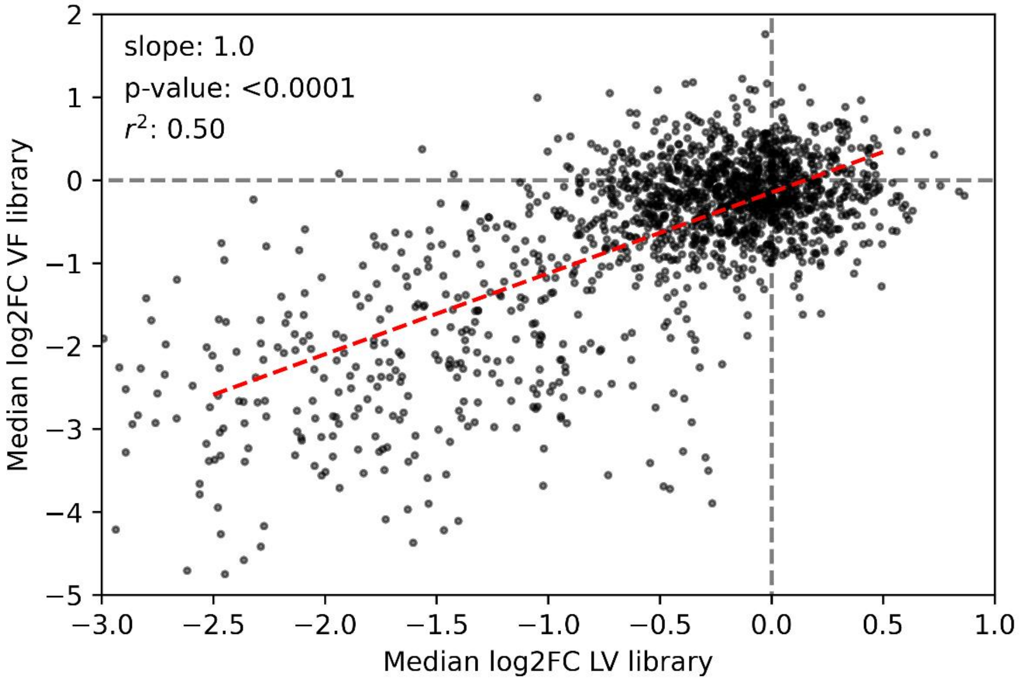

Figure S3 Correlation between virus-free screen and lentiviral based screen. Related to Figure 4

Fold change is calculated with the median (log_2) of gRNA fold change for targeted genes after Cas9 induction in cell pools.

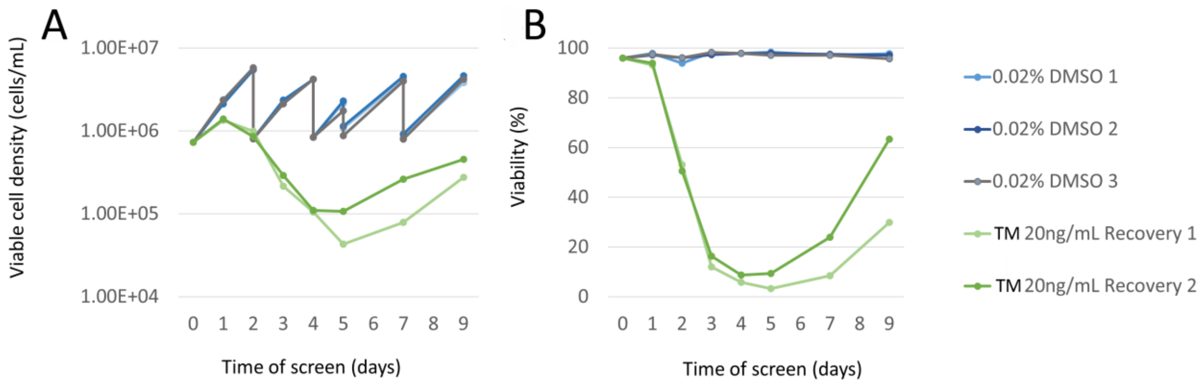

Figure S4 Effects of TM on cell proliferation and viability. Related to figure 5

(**A**) Viable cell density and (**B**) viability of different treated VF CRISPR KO cell pools during TM screen. Established VF CRISPR KO cell pools in duplicates were treated with 20ng/mL of TM for 4 days and recovery for an additional 5 days. Cell pools treated with 0.2% DMSO in triplicates were used as a control. Cells in control groups would be re-seeded to $1x10^6$ cells/mL if the viable cell density reached or nearly reach $5x10^6$ cells/mL

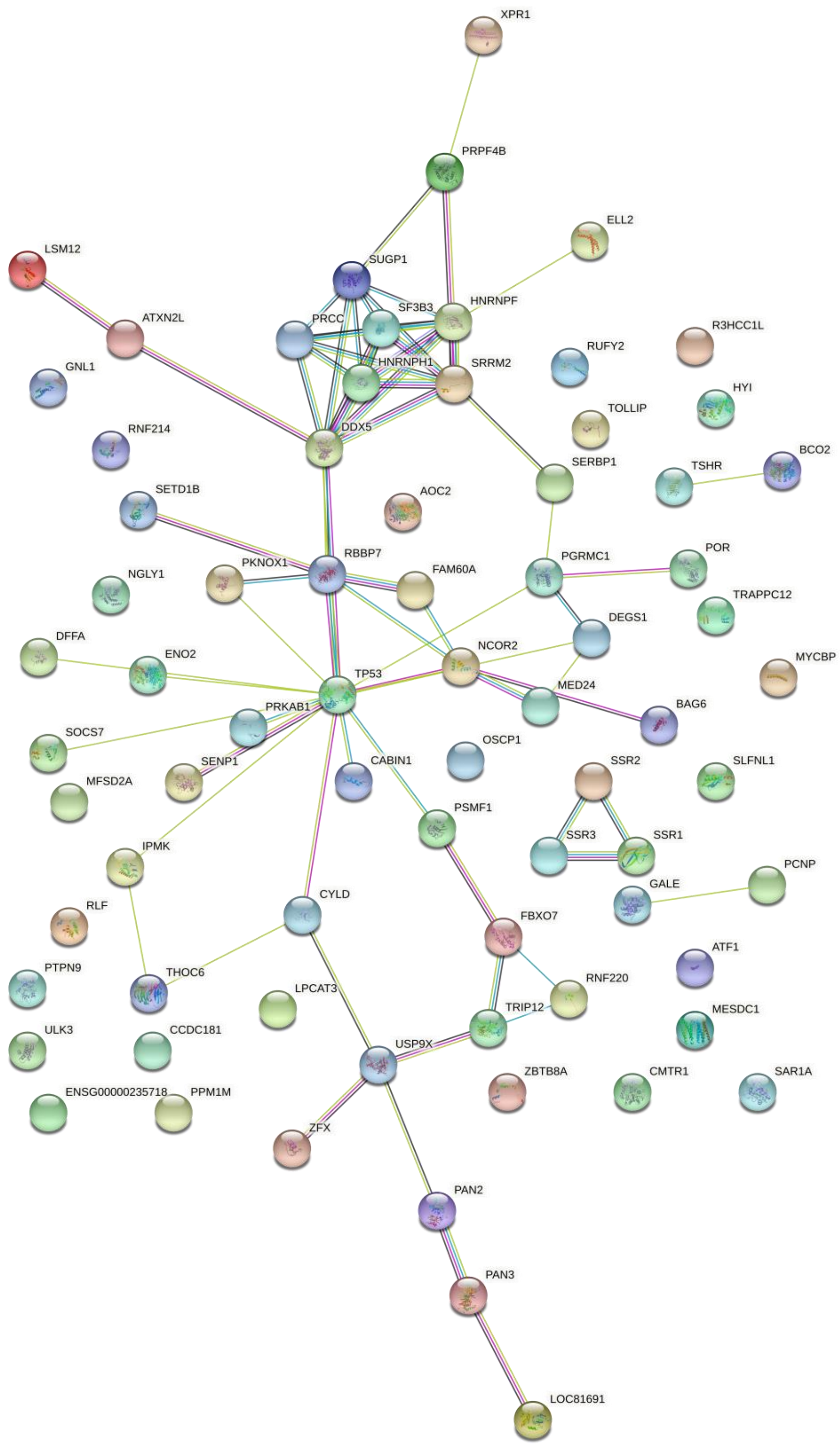

Figure S5 Protein-protein-interaction analysis. Related to figure 5.

Protein-protein-interaction (PPI) analysis in STRING database showing the network of candidate genes whose KO would provide TM resistance in CHO cells. PPI enrichment p-value = 7.42e-08. p<0.05 means that the input proteins have more interactions among themselves than what would be expected for a random set of proteins of similar size, drawn from the genome. Such an enrichment indicates that the proteins are at least partially biologically connected, as a group.

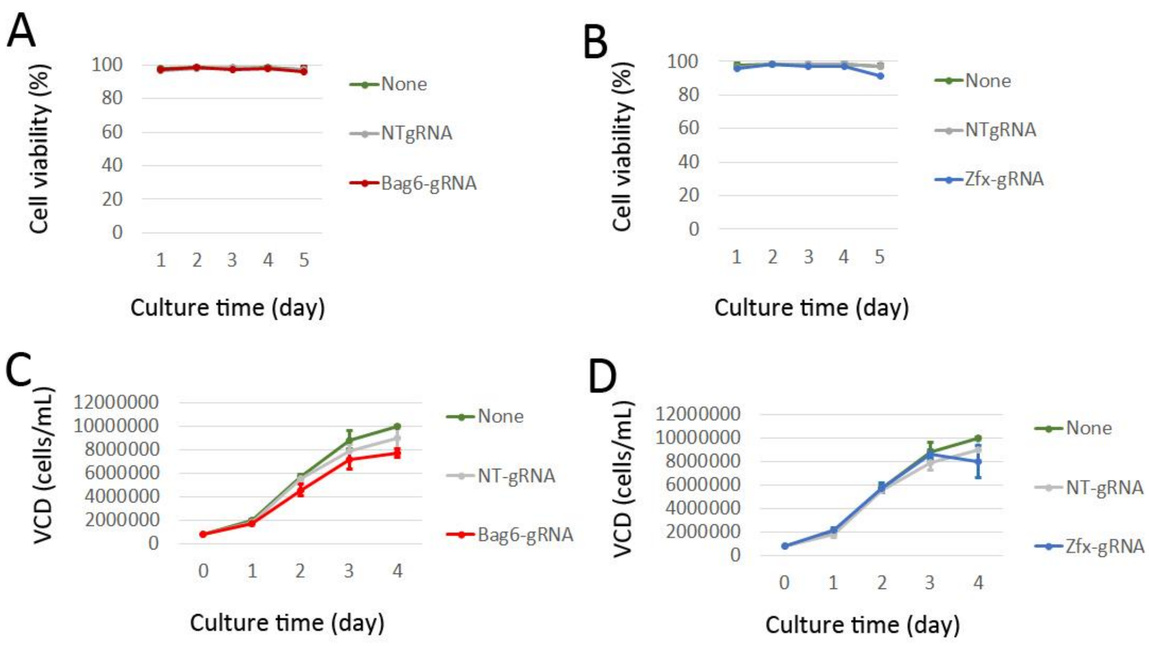

Figure S6 Effect of Bag6 or Zfx KO on cell proliferation and viability. Related to figure 7.

Effects of gRNAs transfection in CHO-Cas9 cells (continuously expressing Cas9) on cell viability (**A, B**) and viable cell density (VCD, **C, D**) targeting (**A, C**) *Bag6* and (**B, D**) *Zfx*. 7 days after transfection of gRNA, the viability and VCD were recorded for 4 days.

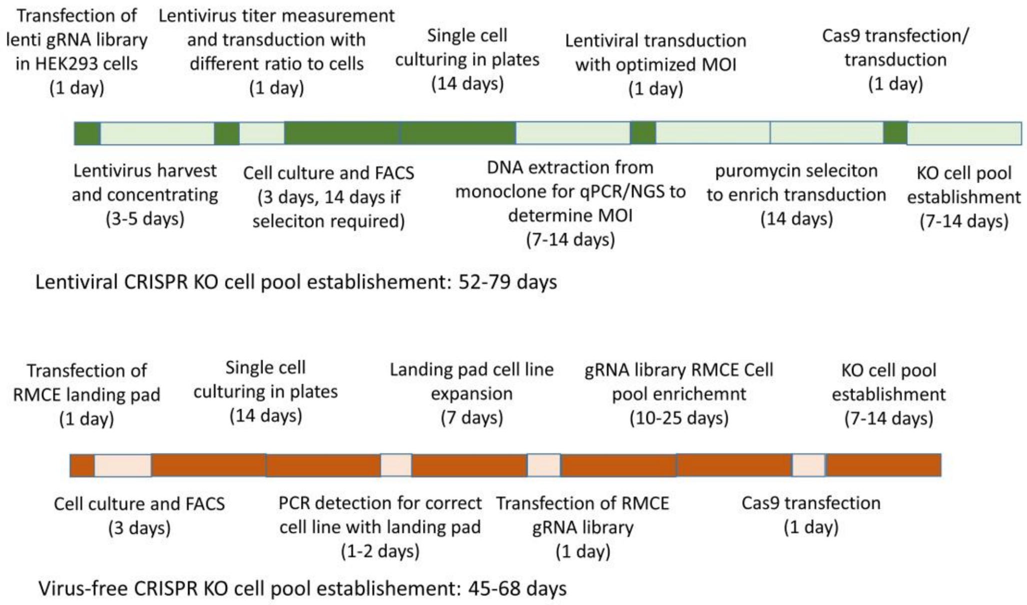

Figure S7 Timeline comparison. Related to figure 1

Timeline comparison between establishing VF CRISPR screen platform and Lentiviral CRISPR screen.
